# Xuetonglactones A–F: Highly Oxidized Lanostane and Cycloartane Triterpenoids From *Kadsura heteroclita* Roxb. Craib.

**DOI:** 10.3389/fchem.2019.00935

**Published:** 2020-01-21

**Authors:** Nuzhat Shehla, Bin Li, Liang Cao, Jianping Zhao, Yuqing Jian, Muhammad Daniyal, Atia-tul Wahab, Ikhlas A. Khan, Duan-fang Liao, Atta-ur Rahman, M. Iqbal Choudhary, Wei Wang

**Affiliations:** ^1^TCM and Ethnomedicine Innovation and Development International Laboratory, Academician Atta-ur-Rahman Belt and Road Traditional Medicine Research Center, School of Pharmacy, Hunan University of Chinese Medicine, Changsha, China; ^2^International Center for Chemical and Biological Sciences, H. E. J. Research Institute of Chemistry, University of Karachi, Karachi, Pakistan; ^3^National Center for Natural Products Research, Research Institute of Pharmaceutical Sciences, University of Mississippi, Oxford, MS, United States; ^4^Dr. Panjwani Center for Molecular Medicine and Drug Research, International Center for Chemical and Biological Sciences, University of Karachi, Karachi, Pakistan

**Keywords:** xuetonglactones, highly oxidized, lanostane triterpenoids, *Kadsura heteroclita*, cytotoxicity

## Abstract

Xuetonglactones A–F (**1**–**6**), six unreported highly oxidized lanostane- and cycloartane-type triterpenoids along with 22 known scaffolds (**7**–**28**) were isolated from the stems of *Kadsura heteroclita* (Roxb.) Craib. Compared with previous congeners, xuetonglactone A (**1**), possesses an unprecedented 20,21-α-epoxide, and xuetonglactone D (**4**) features an unusual 19-α-hydroperoxyl moiety. The structures and the absolute configurations of the compounds were established by extensive one- and two-dimensional NMR, and electronic circular dichroism (ECD) spectroscopic analysis, with those of **1** and **5** confirmed by single-crystal X-ray diffraction technique. Compounds **1** and **2** exhibited inhibition of iNOS activity in LPS-induced macrophages with IC_50_ values of 22.0, and 17.0 μg/mL, respectively. While compounds **6**, **7**, **8**, and **24** showed potent cytotoxic activities against human cervical cancer cell lines (HeLa) with the IC_50_ values of 4.0, 5.8, 5.0, and 6.4 μM, and against human gastric cancer cells (BGC 823) with the IC_50_ values of 2.0, 5.0, 2.5, and 2.0 μM, respectively. Moreover, plausible biogenetic pathways of (**1**–**6**) were also proposed.

**Graphical Abstract:**
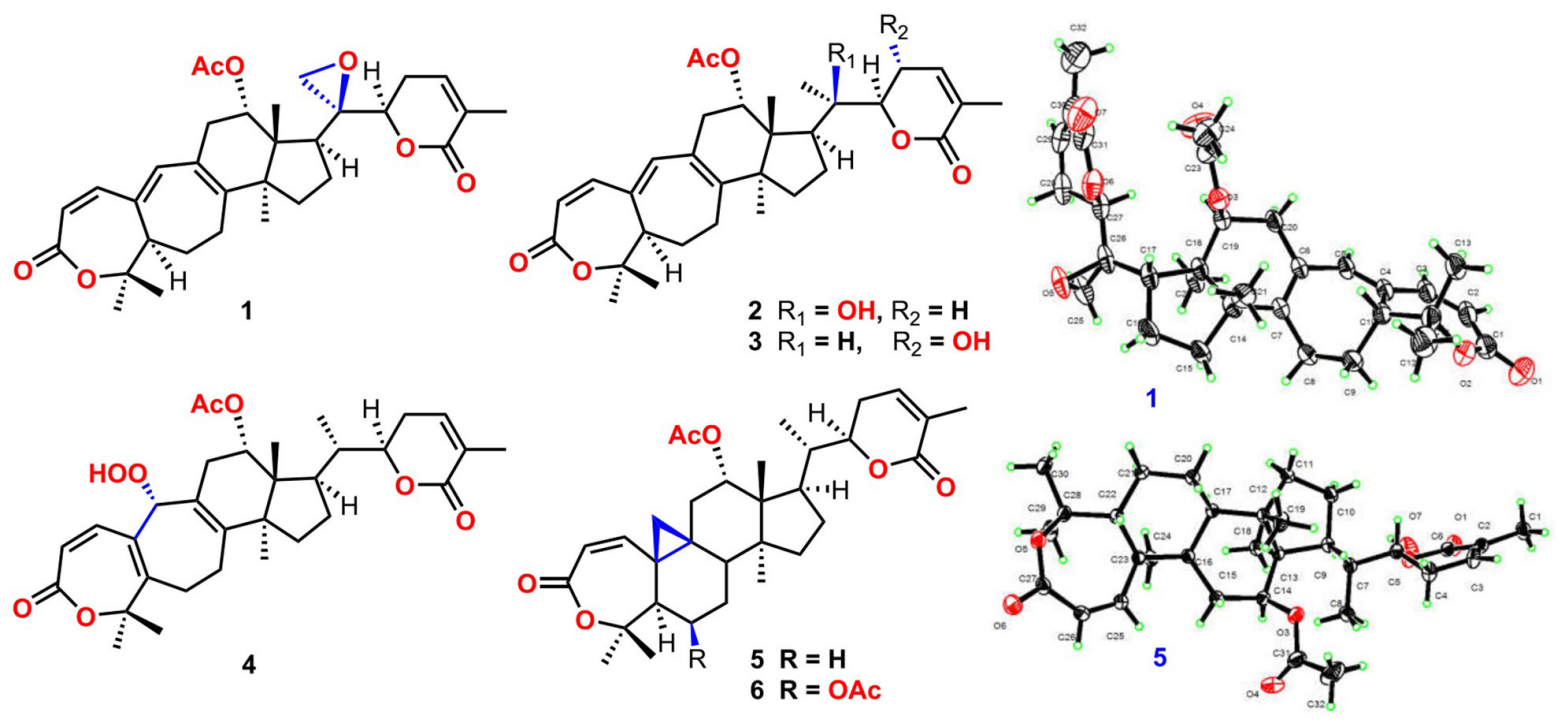
Structures of **1–6**.

## Introduction

Schisandraceae family has contributed to the novel chemical scaffolds with an array of biological activities in past three decades. The family comprises of around fifty plant species belongs to genus *Kadsura* and *Schisandra* that are widely distributed in East, and Southeast Asia. The family has derived significant interest due to its highly oxygenated lanostane- and cycloartane-type triterpenoids, and dibenzocyclooctadiene lignans, along with schinortriterpenoids which are the characteristics isolates (Xiao et al., 2008; Shi et al., 2015). These constituents demonstrated potential pharmacological effects e.g., anti-hepatitis, anti-HIV, anti-inflammatory, anti-cancer, and inhibitory effect in cholesterol biosynthesis (Pu et al., 2008; Liu et al., 2014; Hu et al., 2015; Su et al., 2015).

*Kadsura heteroclita* (Roxb.) Craib. of the genus *Kadsura* is a climbing species primarily grows in Southwestern China, has a long history of its folk use in Traditional Chinese Medicine (TCM) (Pu et al., 2008; Liu et al., 2014). The stems of *K. heteroclita* traditionally known as “Xuetong” has long been consumed for the treatment of rheumatoid arthritis, traumatic injuries, deudenal ulcers, and cancers, particularly by Tujia people living in Wulin mountains area. In “Tujia” dialect “Xue” (blood) herbs are commonly used for the treatment of these diseases by activating the blood circulation, relieving pain and eliminating dampness for centuries (Liu et al., 2016, 2018; Cao et al., 2019). This study aimed to trace back the biologically active chemical constituents responsible for its clinical application contained within the plant species. Recently, we reported the identification of several new sesquiterpenoids, and lignanoids from *K. heteroclita* and other species of the same genus (Liu et al., 2018; Cao et al., 2019). Our previous pharmacological studies displayed this plant has very good anti-rheumatoid arthritis, anti-inflammatory, and analgesic effects (Yu H. et al., 2019; Yu H. H. et al., 2019).

In course of our continuous efforts to crack the immense diversity in structural frameworks with untapped biological potential, herein four new lanostane-type triterpenoids xuetonglactones A–D (**1**–**4**), and two cycloartane-type triterpenoids xuetonglactones E–F (**5**–**6**) possessing differently highly oxidized sites, were reported from *K. heteroclita*. Structurally, xuetonglactones A and D (**1**, **4**) exhibited unique oxidized functionalities, featuring unprecedented 20,21-α-epoxy group in xuetonglactone A, and rare 19-α hydroperoxyl moiety in xuetonglactone D skeletons. The compounds were also evaluated for their cytotoxicity and anti-inflammatory activities. Hence, in this report, the details of isolation, structure elucidation, biological evaluation, and possible biosynthetic pathways of (**1**–**6**) were described. The spectroscopic data of **1**–**6** is presented in the Supplementary Material (Figures S1–S48).

## Results and Discussion

**Figure 1:**
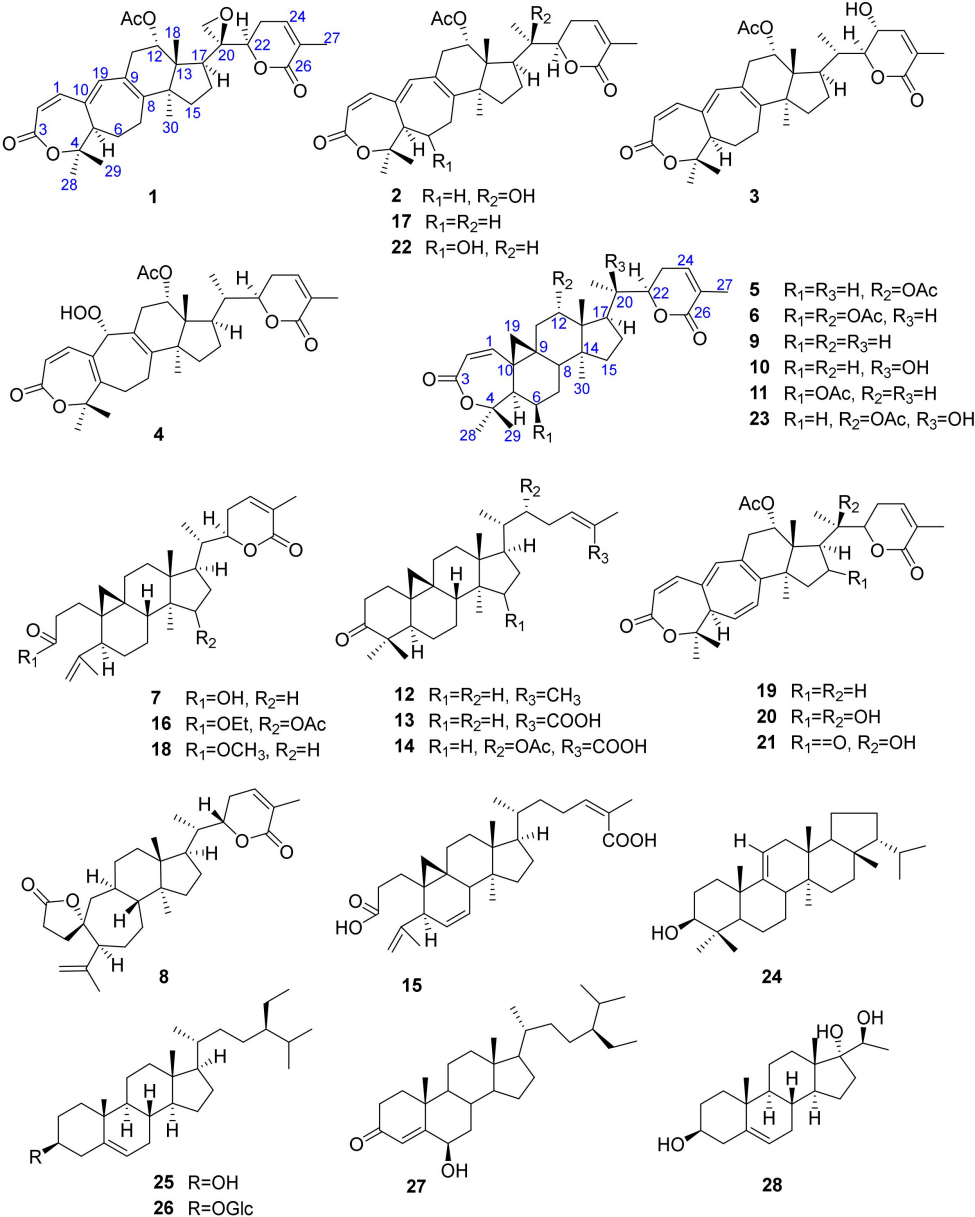
Structures of **1**–**28**.

**Figure 2:**
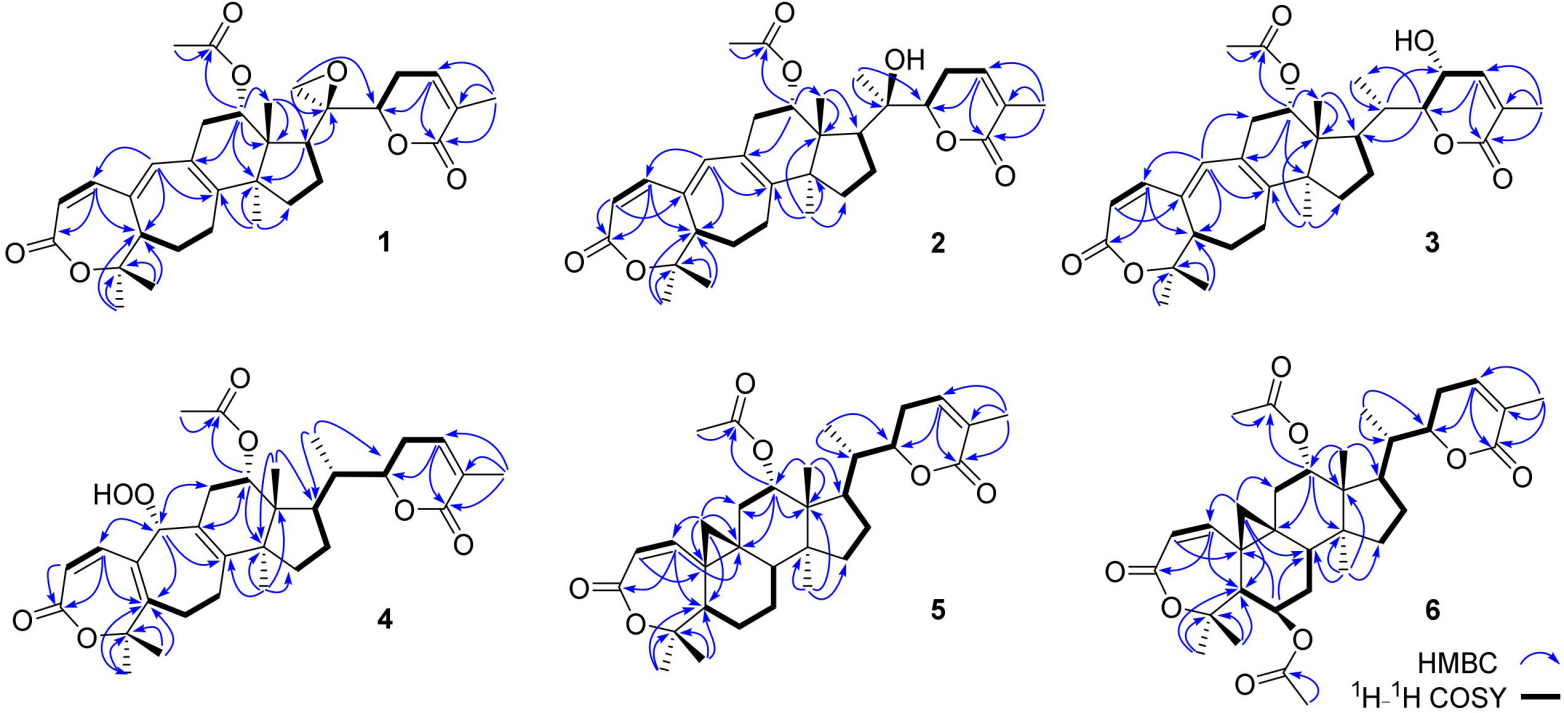
Key HMBC and ^1^H-^1^H COSY correlations of **1**–**6**.

**Figure 3:**
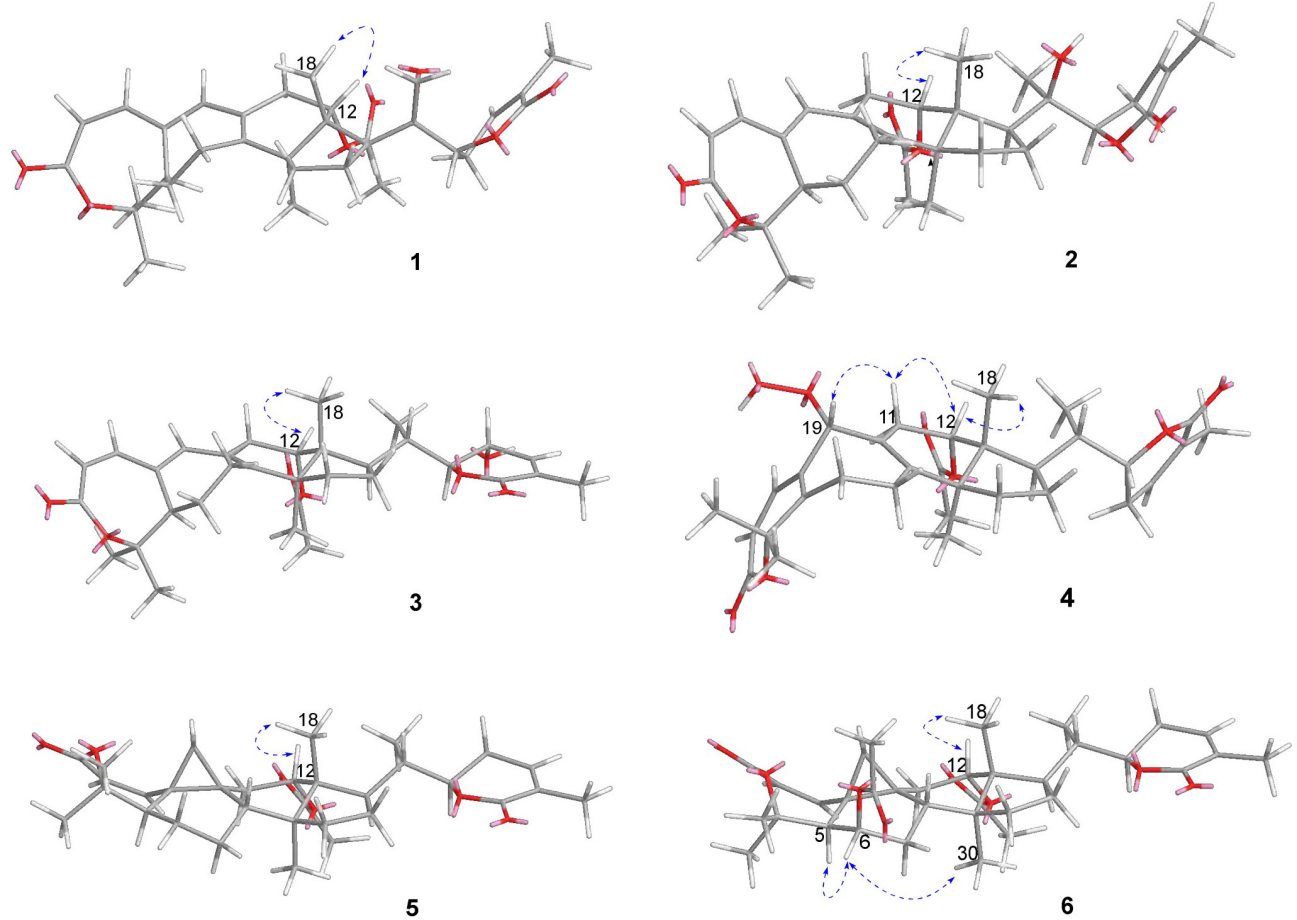
Key ROESY/NOESY correlations of **1**–**6**.

**Figure 4:**
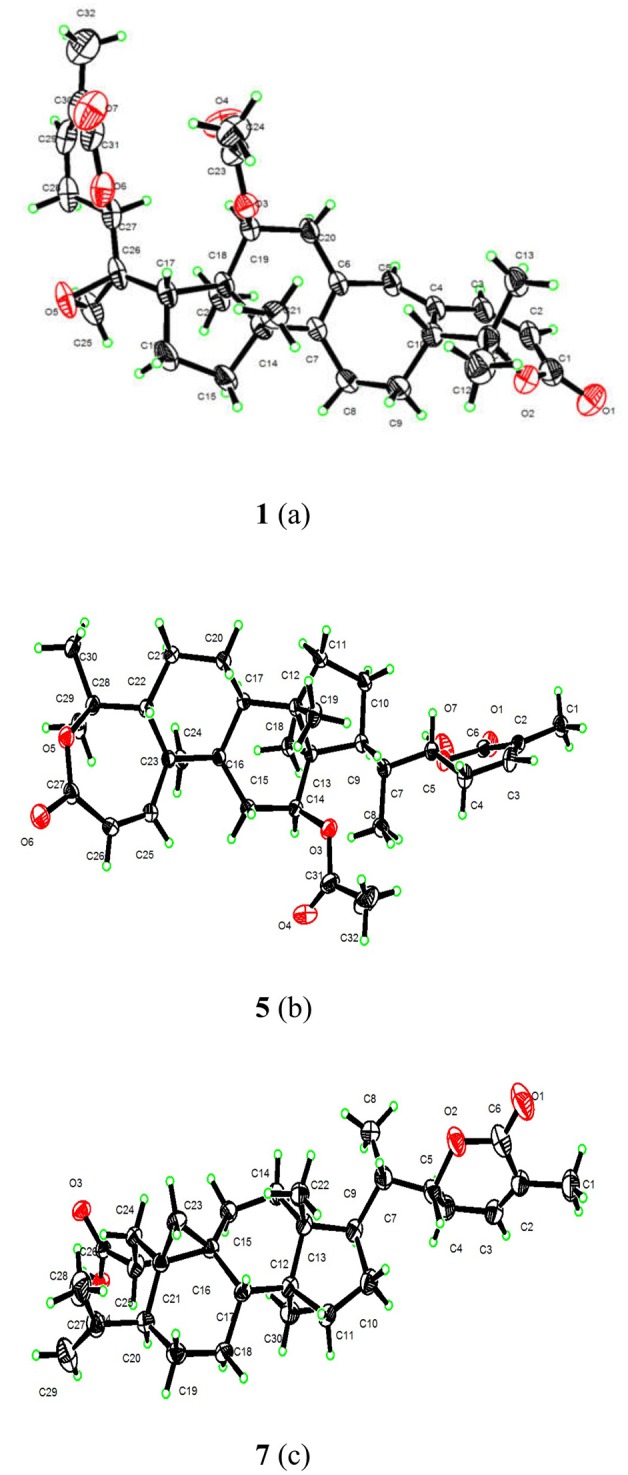
X-ray structures of **1**(a), **5**(b), and **7**(c).

Compound **1** was purified as a white crystalline solid, and the molecular formula was deduced to be C_32_H_40_O_7_ from HRESI-MS spectrum (positive ion mode) on the basis of [M + Na]^+^ ion at *m/z* 559.2671 (559.2672 calculated for C_32_H_40_O_7_ + Na) indicating 13 degrees of unsaturation. A 3,4-secocyclolanostane skeleton was deduced from the ^1^H- and ^13^C-NMR chemical shifts data with two α, β-unsaturated lactone rings, one of them being a seven membered ring in this triterpenoidal skeleton. This deduction was also supported by IR absorptions at 1,720, and 1,685 cm^−1^ for six- and seven-membered unsaturated lactone carbonyls and by UV absorptions (λ_max_) at 202, and 329 nm, respectively. The ^1^H-NMR data of **1** (Table 1) showed the presence of six tertiary methyl singlets (3H each, δ_H_ 0.71, 1.90, 1.53, 1.41, 1.39, and 2.12), four olefinic methines at δ_H_ 6.65 (d, *J*_1, 2_ = 12.3 Hz, H-1), 5.82 (d, *J*_2, 1_ = 12.0 Hz, H-2), 6.14 (s, H-19), and 6.51 (broad d, *J*_24, 23_ = 4.8 Hz, H-24), and two oxygenated methines at δ_H_ 5.14 (d, *J*_12β, 11_ = 7.4, H-12), and 4.49 (dd, *J*_22, 23β_ = 12.7 Hz; *J*_22, 23α_ = 3.7 Hz, H-22). The ^13^C-NMR data (Table 2) displayed 32 carbon signals attributed to six tertiary methyls (δ_C_ 18.6, 16.9, 26.2, 29.3, 27.9, and 21.6), eight methines including four olefinic (δ_C_ 143.2, 118.3, 141.7, and 137.6), and two oxygenated methines (δ_C_ 71.9, and 78.3), seven methylenes, and eight quaternary carbons, including four olefinic (δ_C_ 151.1, 126.4, 140.4, and 128.2), and two oxygenated (δ_C_ 80.3, and 58.1) quaternary carbons. Furthermore, it also showed the presence of three carbonyl signals (δ_C_ 171.0, δ_C_ 166.3, and δ_C_ 165.0) corresponding to an acetoxy (C-12), and two lactone (C-3, and C-26) moieties, respectively. The ^1^H- and ^13^C-NMR chemical shifts data of **1** showed resemblances with that of known compound heteroclitalactone D (**17**) (Wang et al., 2006b) with obvious distinctions observed for resonances at C-17, C-20, and C-21. The detailed analysis of the NMR data established the structure of compound **1** bearing unprecedented oxirane in the structure at C-20. The NMR data revealed the presence of an extra methylene (δ_C_ 46.88, C-21) with a characteristic proton doublet (δ_H_ 2.75; and 2.96, dd, *J*_21a, 21b_ = 3.3 Hz each, H-21) which supported the presence of additional ring at C-20 as epoxide to fulfill the unsaturation demand in skeleton of **1**. Furthermore, the up-field shift of quaternary carbon (δ_C_ 58.12) at C-20 suggested the presences of epoxide at this junction, which could be attributed to the steric shielding effect of the strained ring at this position. These assignments were unambiguously confirmed by HMBC experiments, in which the epoxide methylene protons appeared at δ_H_ 2.75, and 2.96 (dd, *J*_21a, 21b_ = 3.3 Hz each, H-21) were correlated with C-20 and C-22, while C-17 methine proton at δ_H_ 3.55 (dd, *J*_17, 16a_ = 10.9, *J*_17, 16b_ = 7.5 Hz) was correlated with C-12, C-14, C-16, C-18, C-20, and C-21 (**Figure 2**). This oxidized strained ring along with the presence of three conjugated double bonds, and other oxygenated moieties in the skeleton further supported the structure of **1** is based on a highly oxidized cyclolanostane type triterpenoidal skeleton (Chen et al., 2001; Wang et al., 2006a). Chemical shifts assignments were made on the basis of HSQC, HMBC, and ^1^H-^1^H COSY experiments to get the planner structure of **1**. The α-configuration of the C-12 (acetoxy group) was concluded based on the ROESY cross peaks between H-12 and CH_3_-18 (**Figure 3**). In the ECD spectrum, compound **1** showed a positive Cotton effect at 255 nm (Δε = + 5.64) which was similar to that of schiglausin A (Zou et al., 2012), indicating an *R* configuration of C-22. Combining the observed ROESY correlations of H-5 with CH_3_-30, CH_3_-30 with H-17, H-17 with H-22, the ECD spectrum, and the X-ray diffraction using Cu Kα radiation (**Figure 4**), the absolute stereochemistry of the seven chiral centers, were determined as 5*S*, 12*S*, 13*R*, 14*S*, 17*R*, 20*S*, and 22*R*. Thus, the structure of **1** was fully established as shown (Figure 1) and named xuetonglactone A.

**Table 1:** ^1^H NMR data of **1**–**6** in CDCl_3_ (δ_H_ in ppm, *J* in Hz within the parenthesis).

**No**.	**1^a^**	**2^a^**	**3^b^**	**4^b^**	**5^b^**	**6^a^**
1	6.65, d (12.3)	6.66, d (12.3)	6.68, d (12.3)	6.58, d (12.2)	6.05, d (12.7)	6.00, d (12.8)
2	5.82, d (12.0)	5.81, d (12.0)	5.83, d (12.1)	6.21, d (12.0)	5.94, d (12.6)	5.93, d (12.7)
3	–	–	–	–	–	–
4	–	–	–	–	–	–
5	2.44, m	2.48, m	2.47, m	–	2.42, dd (13.0, 4.7); 1.62, m	2.54, d (3.5)
6	2.27, m	2.25, m	3.12, 2.59 m	3.20, t (13.4, 1); 2.34, m	1.90, m; 0.76, m	5.31, brs
7	1.92, m	1.91, m	2.13, 1.94 m	2.04, m; 2.40, m	1.18, m	1.84, m
8	–	–	–	–	1.62, m	1.78, dd (13.5, 4.0)
9	–	–	–	–	–	–
10	–	–	–	–	–	–
11	2.10, m	2.10, m	2.81, m; 2.13, m	2.80, dd (19.6, 8.0); 2.49 d (19.6)	2.00, dd (15.2, 5.2); 2.32, m	2.01, m
12	5.14, d (7.4)	5.31, d (7.2)	5.03, d (7.5)	4.94, d (7.9)	4.85, dd (8.8, 5.3)	4.87, dd (9.2, 5.7)
13	–	–	–	–	–	–
14	–	–	–	–	–	–
15	1.45, m	1.44, m	1.74, 1.44 m	1.67, m; 131, m	1.39, m	1.30, m
16	1.25, m	1.71, m	2.15, 1.53, m	1.89, m; 1.51, m	1.46, m	1.25, m
17	3.55, dd (10.9, 7.5)	2.70, m	2.66, m	2.11, m	2.22, m	2.23, m
18	0.71, s	0.96, s	0.75, s	0.76, s	1.01, s	1.05, s
19	6.14, s	6.16, s	6.18, s	4.61, s	1.15, d (2.6), 1.38 (overlapped)	1.30, dd (4.7)
20	–	–	2.04, m	2.10, m	2.01, m	2.02, m
21	2.75, dd (3.3)	1.33, s	0.98, d (7.0)	0.89, d (6.5)	0.85, d (6.7)	0.86, d (6.7)
22	4.49, dd (12.7, 3.7)	4.14, dd (12.7, 3.8)	4.34, dd (9.4, 2.5)	4.46, d (13.1)	4.48, dt (13.0, 3.2)	4.48, dd (9.8, 3.3)
23	2.03, m	2.32, m	4.56, br. d (7.7)	2.36, m; 2.11, m	2.11, m	2.14, m
24	6.51, dd (4.8, 1.6)	6.60, dd (4.6, 1.7)	6.480, s	6.60, s	6.61, d (6.0)	6.61, d (6.0)
25	–	–	–	–	–	–
26	–	–	–	–	–	–
27	1.90, s	1.91, s	1.93, s	–	1.92, s	1.92, s
28	1.53, s	1.54, s	126, s	1.13, s	1.35, s	1.47, s
29	1.41, s	1.41, s	1.54, s	1.64, s	1.38, s	1.42, s
30	1.39, s	1.32, s	1.41, s	1.67, s	1.01, s	1.03, s
OCOCH_3_-12	2.12, s	2.13, s	2.09, s	2.05, s	2.04, s	2.04, s
OCOCH_3_-6	–	–	–	–	–	2.05, s

a *Recorded at 500 MHz*.

b *Recorded at 600 MHz*.

*Internal standard: TMS; In ^1^H-NMR s, brs, d, dd, and m represent singlet, broad singlet, doublet, double doublet, and multiplet or overlapped signals, respectively*.

**Table 2:** ^13^C-NMR data of **1**–**6** in CDCl_3_ (δ in ppm).

**No**.	**1^a^**	**2^a^**	**3^b^**	**4^b^**	**5^b^**	**6^a^**
1	143.2, d	143.5, d	143.5, d	142.8, d	150.0, d	149.4, d
2	118.3, d	118.1, d	118.1, d	123.7, d	120.9, d	120.5, d
3	166.3, s	167.0, s	167.1, s	167.0, s	167.3, s	166.4, s
4	80.3, s	80.3, s	80.4, s	81.5, s	84.5, s	83.4, s
5	49.2, d	49.2, d	49.2, d	153.1, s	46.7, d	48.1, d
6	39.5, t	39.7, t	37.4, t	26.6, t	24.8, t	70.3, d
7	28.0, t	28.0, t	27.2, t	26.9, t	25.0, t	28.7, t
8	151.1, s	150.9, s	151.4, s	147.5, s	46.8, d	40.7, d
9	126.4, s	126.6, s	126.7, s	121.2, s	27.1, s	29.7, s
10	140.7, s	140.3, s	140.4, s	133.7, s	33.0, s	31.7, s
11	35.4, t	35.2, t	35.2, t	39.2, t	37.5, t	37.4, t
12	71.9, d	74.0, d	73.8, d	74.1, d	75.0, d	74.6, d
13	52.3, s	51.2, s	51.6, s	51.1, s	48.4, s	47.8, s
14	48.5, s	49.3, s	48.2, s	47.8, s	48.8, s	48.5, s
15	31.9, t	32.3, t	32.0, t	30.6, t	36.2, t	36.6, t
16	20.9, t	23.5, t	27.9, t	26.3, t	26.8, t	26.8, t
17	35.5, d	41.2, d	39.4, d	39.3, d	40.1, d	40.0, d
18	18.6, q	18.5, q	16.3, q	16.3, q	16.7, q	16.6, q
19	141.7, d	142.1, d	142.2, d	90.3, d	32.9, t	35.6, t
20	58.1, s	75.9, s	39.7, d	38.9, d	39.1, d	39.0, d
21	46.9, t	21.5, q	13.7, q	12.5, q	12.1, q	120, q
22	78.3, d	84.3, d	84.6, d	80.0, d	80.3, d	80.1, d
23	24.3, t	24.4, t	64.0, d	23.4, t	23.4, t	23.3, t
24	137.8, d	139.0, d	143.7, d	139.0, d	139.3, d	139.0, d
25	128.2, s	128.3, s	127.8, s	128.5, s	128.6, s	128.5, s
26	165.0, s	165.3, s	165.0, s	166.3, s	166.5, s	166.2, s
27	16.9, q	17.0, q	16.7, q	17.0, q	17.2, q	17.0, q
28	26.2, q	26.2, q	27.6, q	28.2, q	22.4, q	24.0, q
29	29.3, q	29.2, q	26.3, q	24.6, q	29.2, q	28.0, q
30	27.9, q	28.2, q	29.2, q	24.6, q	20.5, q	20.6, q
OCOCH_3_-12	171.0, s	171.4, s	170.2, s	170.0, s	170.1, s	169.8, s
OCOCH*_3_*-12	21.6, q	21.8, q	21.4, q	21.3, q	21.5, q	21.1, q
OCOCH_3_-6	–	–		–	–	169.5, s
OCOCH*_3_*-6	–	–		–	–	21.3, q

a *Recorded at 500 MHz*.

b *Recorded at 600 MHz*.

*Internal standard: TMS; In ^13^C-NMR d, t, q, and s represent methine, methylene, methyl, quaternary carbons; Chemical shifts assignments based on DEPT, HSQC, and HMBC data*.

Compound **2** was obtained as a white amorphous solid. Its molecular formula was determined to be C_32_H_42_O_7_ based on [M + Na]^+^ ion at *m/z* 561.2828 (561.2829 calculated for C_32_H_42_NaO_7_) and [M + Cl]^−^ peak at *m/z* 573.2612 (573.2619 calculated for C_32_H_42_O_7_ + Cl) from its HRESI-MS spectra (positive ion and negative ion modes), corresponding 12 degrees of unsaturation. The ^1^H-NMR chemical shifts data (Table 1) of **2** showed the presence of seven tertiary methyl singlets (3H each, δ_H_ 0.96, 1.32, 1.33, 1.41, 1.54, 1.91, and 2.13). By comparing the NMR chemical shifts data of compound **2** with that of **1** which has a methylene at position C-21, hence the presence of a tertiary methyl (δ_H_ 1.33, s, C-21) instead of epoxide, in the skeleton of **2** was inferred. Furthermore, relative configuration on ring C was same as of **1**, based on similar ROESY correlations. While the absence of ROESY cross peaks between CH_3_-21 and CH_3_-18 thus appearance of cross peaks between CH_3_-21 and H-17/H-22 (**Figure 3**) suggested the β-orientation of OH-20. The assignment of the absolute configuration at C-22 was concluded to be *R* by the similar ECD measurement as that of **1**. Therefore, the structure of **2** was established as shown (Figure 1) and named xuetonglactone B accordingly.

Compound **3** was obtained as a white amorphous solid and possess the same molecular formula with **2** as C_32_H_42_O_7_ based on [M + Na]^+^ ion at *m/z* 561.2836 (561.2828 calculated for C_32_H_42_O_7_ + Na) from its HRESI-MS spectrum (positive ion mode). The spectroscopic data of **3** (Tables 1, 2) was quite similar with those of **2** except for a secondary methyl (δ_H_ 0.98, *J*_21, 20_ = 7.0 Hz, H-21) instead of tertiary, and a broad doublet of oxymethine (δ_H_ 4.56, *J*_23, 22_ = 7.7 Hz, δ_C_ 64.0, H-23) instead of methylene protons in the skeleton of **3**. These observations were also confirmed by HMBC cross peaks of H-20 with C-23, and that of H-22 with C-17, C-23, and C-21, and C-24. Furthermore, α-configuration of the acetoxy group was determined by the similar cross peaks between H-12 and CH_3_-18 in NOESY spectrum (**Figure 3**). A strong negative Cotton effect at 273 nm [Δε (273) = +0.41, MeOH] was observed in the experimental ECD spectrum of **3**. The absolute configuration at C-22 was established as *S* by comparison of its ECD spectrum with analog colossolactone VIII (El Dine et al., 2008). Furthermore, the large coupling constant (9.4 Hz) between H-23 and H-22 indicated an anti-conformation of these two protons (Lakornwong et al., 2014), hence the absolute configuration of these two chiral centers were found to be 22*S*, and 23*R*. Consequently, compound **3** was determined and given the trivial name xuetonglactone C.

Compound **4** was isolated as yellow, amorphous solid. Its molecular formula was deduced as C_32_H_42_O_8_ on the basis of [M + Na]^+^ ion peak at *m/z* 577.2772 in the HRESI-MS (577.2777 calculated for C_32_H_42_O_8_ + Na), suggesting 12 degrees of unsaturation. Since the NMR resonances of **4** were similar to those of known compound **17** (Wang et al., 2006b) with some obvious discrepancies, therefore detailed comparison of the chemical shifts data revealed that both compounds have similar C/D/E rings system in the skeleton. However, different ^1^H- and ^13^C-NMR chemical shifts were observed for C-5, C-6, C-10, and C-19 suggesting the major skeletal difference of **4** corresponds to the rings A/B. The double bond between C-10 and C-19 in **17** shifted in between C-5 (δ_C_ 153.1) and C-10 (δ_C_ 133.7) in **4**. This inference was further supported by the HMBC correlation of H-1 (δ_H_ 6.58, d, *J*_1, 2_ = 12.2 Hz), H-19 (δ_H_ 4.61, s), H_3_-29 (δ_H_ 1.64, s), and H_3_-30 (δ_H_ 1.67, s) with C-5, and of H-2 (δ_H_ 6.21, d, *J*_2, 1_ = 12.0 Hz) and H-19 with C-10. Furthermore, C-19 was connected with an unusual hydroperoxyl group, which was supported by the carbon resonance observed at δ_C_ 90.3 and the methine proton signal at δ_H_ 4.61 (1H, s) (Song et al., 2013), which was also confirmed by HMBC cross peaks of H-1 and H-11 to C-19, and H-19 to C-1, C-5, C-8, C-9, C-10, and C-11. α oriented 12-acetoxyl group was ascertained on the basis of NOESY cross peak between H _β_-12 and CH_3_-18, the significant NOESY correlations between H_β_-12 /H_β_-11 (δ _H_ 2.82, *J*_Hβ−11/*Hα*−11_ = 19.6, *J*_Hβ−11/*Hβ*−12_ 8.0 Hz), and H_β_-11/H_β_-19 indicated that the hydroperoxyl group should be α-orientated (**Figure 3**). The absolute configuration 22*R* could be delineated by similar ECD relationship (Wang et al., 2006b). Hence the structure of **4** was determined and given a trivial name xuetonglactone D accordingly.

Compound **5** was obtained as white crystalline. The HRESI-MS spectrum of **5** displayed [M + Na]^+^ ion peak at *m/z* 547.3047 (547.3036 calculated for C_32_H_44_O_6_ + Na), corresponding to the molecular formula of C_32_H_44_O_6_ indicative of 11 degrees of unsaturation. The IR spectrum showed absorptions at 1,721, and 1,679 cm^−1^ suggesting two lactone moieties in the skeleton. A 3,4-secocycloartane skeleton was deduced from ^1^H- and ^13^C-NMR chemical shifts data (Tables 1, 2). The ^1^H-NMR spectrum of **5** showed characteristic signals for the cyclopropyl methylene protons at δ_H_ 1.15 (d, *J*_19a, 19b_ = 2.6 Hz), and 1.38 (overlapped), but unlike the known compound **7** (Liu and Huang, 1991), the downfield shift of cyclopropane protons was due to the deshielding effect of conjugated double bond in ring A. The ^1^H-NMR also displayed six tertiary methyl singlets (δ_H_ 1.01, 1.92, 1.35, 1.38, 1.01, and 2.04), and a secondary methyl proton at δ_H_ 0.85 (d, *J*_21, 20β_ = 6.7 Hz, H-21). In ^13^C-NMR spectrum presence of 32 carbon signals (Table 2) could be assigned to six tertiary (δ_C_ 16.7, 17.2, 22.4, 29.2, 20.5, and 21.5), and a secondary methyl (δ_C_ 12.1), ten methines, seven methylenes, and nine quaternary carbons including three carbonyl (δ_C_ 167.3, 166.5, and 170.1), an oxygenated (δ_C_ 84.5), and an olefinic quaternary carbons (δ_C_ 128.6), as well as two oxygenated (δ_C_ 75.0, and 80.3), and three olefinic methine (δ_C_ 150.0, 120.9, and 139.3) signals. In comparison of **3**, the major difference in ^1^H- and ^13^C-NMR chemical shifts data of **5** was obvious owing to absence of a pair of olefinic carbons and a proton in ring B, thus appearance of a C-19 cyclopropane ring proton doublets (δ_H_ 1.15, d, *J*_19a, 19b_ = 2.6 Hz), and 1.38 (overlapped) corresponding to δ_C_ 32.9 instead in the structure. These distinctions were confirmed by the key correlations observed in ^1^H-^1^H COSY, and HMBC spectra. In HMBC spectrum CH_2_-19 protons correlated with C-1, C-10, C-5, C-8, C-9, and C10. Additionally, the ^1^H-^1^H COSY interactions between H-11/H12, H-20/H-21, H-20/H-22, H-22/H-23, and H-23/H-24 spin systems were consistent with the unambiguous spectral assignments based on HSQC, and HMBC interactions (Figure 2). Moreover, α-configurations of 12-OCOCH_3_ was determined by ROESY spectrum (Figure 3). Since a strong positive cotton effect at 272 nm (Δε = +3.31, MeOH) was observed, the absolute configuration at C-22 in **5** was consequently assigned as *R*-configuration by ECD measurement. The absolute configuration at C-22 in **5**, named xuetonglactone E was also further confirmed by single crystal X-ray diffraction technique (Figure 4).

Compound **6** was purified as white amorphous solid. The molecular formula was assigned as C_34_H_46_O_8_ based on molecular ion peak at 617.2884 m/z [M + Cl]^−^ (617.2881 calculated for C_34_H_46_O_8_ + Cl) in the HRESI-MS spectrum, which was indicative of 12 unsaturation degrees. The ^13^C-NMR spectrum of **6** exhibited 34 carbon resonances, attributed to seven tertiary, and a secondary methyls, 10 methines, 6 methylenes, and 10 quaternary carbons. Four ester carbonyls (δ_C_ 166.4, 166.2, 169.5, 169.8, and) corresponding to C-3, C-26, OCOCH_3_-6, and OCOCH_3_-12, respectively were observed in **6**. The ^1^H- and ^13^C-NMR chemical shifts data of **6** (Tables 1, 2) were extremely similar to those of **5**, and the major difference embodied in the chemical shift of C-6 (δ_H_ 5.31, δ_C_ 70.3) suggesting the appearance of an additional acetyl group at C-6 [δ_H_ 2.05 (δ_C_ 21.3, 169.5)], thus absence of a methylene, and appearance of an oxygenated methine in **6**. Furthermore, configurations of 6-OCOCH_3_ and 12-OCOCH_3_ were determined based on the ROESY cross peaks of H-5/H-6/CH_3_-30/ and H-12/CH_3_-18, so β- 6-OCOCH_3_ and α-12-OCOCH_3_ were inferred (Figure 3). The ECD spectrum of **6** was the same with that of **5**, hence C-22 was assigned to the *R*-configuration, thus the absolute structure was determined as shown (Figure 1), and named xuetonglactone F accordingly.

**Figure 5:**
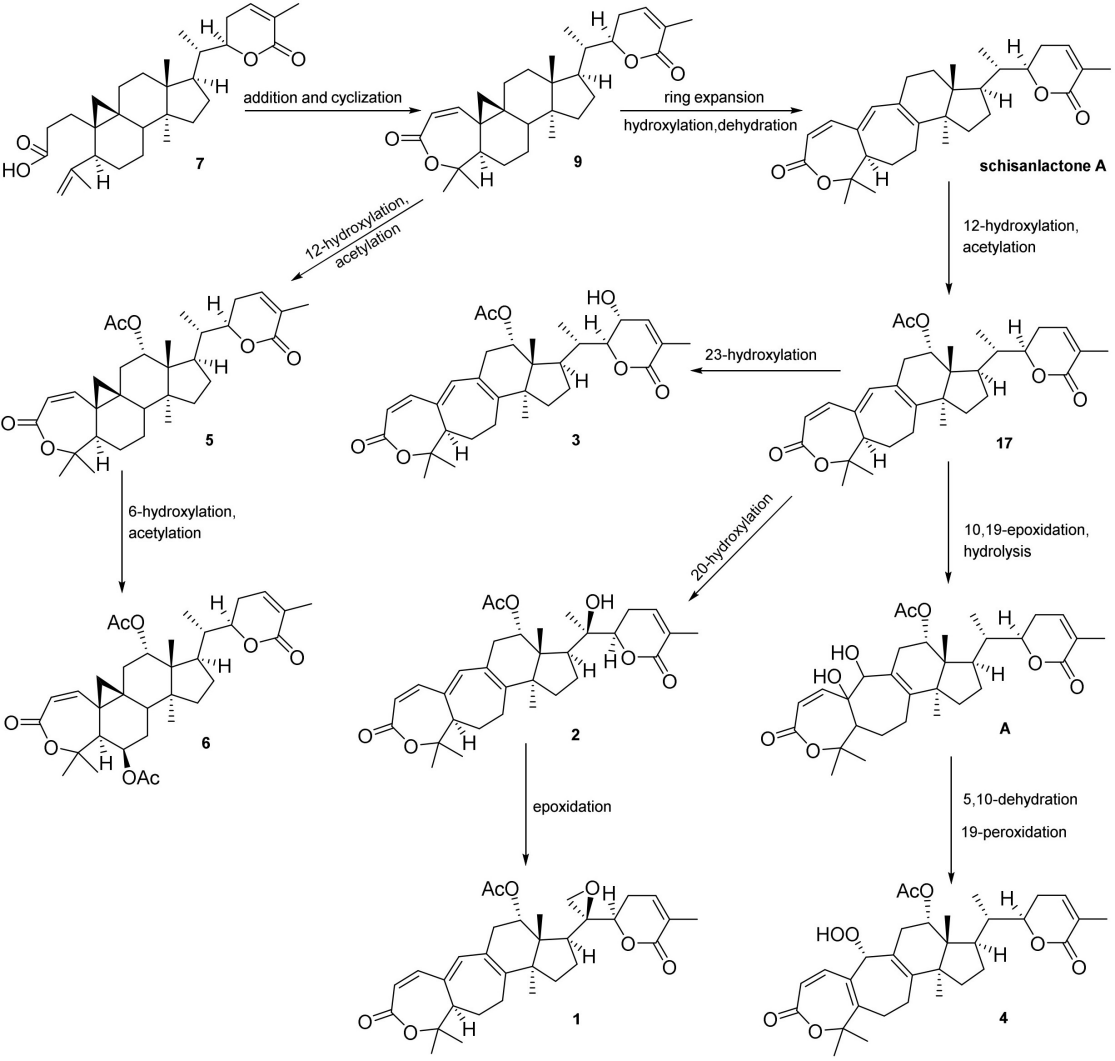
Plausible biosynthetic pathway for **1**–**6**.

Xuetongsu (schisanlactone E, **7**) (Liu and Huang, 1991) was isolated as colorless crystal. The X-ray diffraction data of **7** was reported for the first time in this report (Figure 4). It was the major compound in “Xuetong” (Wang et al., 2006c). Biosynthetically, it might be the precursor of compounds **1**–**6**, through series of oxidative cleavage *via* Baeyer-Villiger oxidation, ring expansion, hydroxylation, cyclization, acetoxylation, and epoxidation steps yielded compounds **1**–**6**. A plausible biogenetic route for **1**–**6** was proposed as shown in Figure 5.

Twenty-two known analogous (**7**–**28**) were identified by analysis of their spectroscopic data with the reported data for xuetongsu (schisanlactone E, **7**) 10.8 g (Liu and Huang, 1991), kadnanolactone A (**8**) 5.0 mg (Yang et al., 2010), schisanlactone B (**9**) 25.0 mg (Liu et al., 1983), kadsuphilactone B (**10**) 6.8 mg (Shen et al., 2005), schisanbilactone A (**11**) 4.0 mg (Ma et al., 2009), cycloartenone (**12**) 2.5 g (Pavanasisivam and Sultanbawa, 1973), schisandronic acid (**13**) 18.2 mg (Li et al., 2003), heteroclic acid (**14**) 5.6 mg (Wang et al., 2006b), changnanic acid (**15**) 18.0 mg (Liu and Huang, 1991), heteroclitalactone C (**16**) 5.5 mg (Wang et al., 2006b), heteroclitalactone D (**17**) 42.0 mg (Wang et al., 2006b), heteroclitalactone F (**18**) 7.0 mg (Wang et al., 2006b), heteroclitalactone G (**19**) 22.0 mg (Wang et al., 2007), heteroclitalactone I (**20**) 6.5 mg (Wang et al., 2007), heteroclitalactone K (**21**) 12.5 mg (Wang et al., 2007), heteroclitalactone L (**22**) 15.3 mg (Wang et al., 2007), heteroclitalactone M (**23**) 17.3 mg (Wang et al., 2007), sorghumol (**24**) 12.9 mg (Han et al., 2008), β-sitosterol (**25**) 100 mg (Chaturvedula and Prakash, 2012), daucosterol (**26**) 10.0 mg (Rahmana et al., 2009), 6β-hydoxysitostenone (**27**) 7.6 mg (Liang et al., 2015), and a steroid, trihydoxy pregnene (**28**) 13.0 mg (Deng et al., 2010). Their structures are presented in Figure 1.

Anti-inflammatory activity of the compounds **1**–**6** were evaluated for their inhibitory effects against iNOS, and NF-κB activation. Compounds **1** and **2** showed inhibition of iNOS activity in LPS-induced macrophages with the IC_50_ values of 22.0, and 17.0 μg/mL (Table 3), respectively, while parthenolide was used as control drug (Table 3), unfortunately no inhibitory effects found against NF-κB expression (Zhao et al., 2014). Additionally, cytotoxic activities of all the compounds against HeLa and BGC-823 cancer cell lines were also evaluated (Table 4). Compounds **6**, **7**, **8**, and **24** showed strong cytotoxicities against HeLa cancer cell lines with the IC_50_ values of 4.0, 5.8, 5.0, and 6.4 μM, and against BGC 823 with the IC_50_ values of 2.0, 5.0, 2.5, and 2.0 μM, respectively, while compared with paclitaxel as positive control (Table 4; Hayon et al., 2003).

**Table 3:** Inhibition of iNOS activities of the tested compounds.

**Compounds**	**IC_**50**_ (μM)**
**1**	22.0
**2**	17.0
**6**	NA
Parthenolide	3.2

*NA, Not active*.

**Table 4:** Cytotoxicities of the tested compounds on HeLa and BCG-823 cancer cell lines.

**Compounds**	**HeLa** ** IC_**50**_ (μM)**	**BCG-823** ** IC_**50**_ (μM)**
**2**	48.22	24.38
**5**	38.92	33.28
**6**	4.0	2.0
**7**	5.8	5.0
**8**	5.0	2.5
**10**	35.25	25.98
**14**	34.56	22.93
**15**	38.89	27.17
**20**	33.23	20.22
**21**	45.87	21.032
**22**	29.89	18.47
**23**	50.23	21.51
**24**	6.4	2.0
Paclitaxel	0.0026	0.010

## Materials and Methods

### General Experimental Procedure

Optical rotations were measured on a PerkinElmer 341-MC digital polarimeter, UV spectra were recorded on a TU-1900 spectrophotometer; A Hitachi 260-30 spectrometer was used for scanning IR spectroscopy; Experimental ECD spectra were recorded on a JASCO J-815 Circular Dichroism (CD) Spectropolarimeter; NMR spectra were performed on Bruker ARX-600 spectrometers, and on Agilent DD2-500 NMR spectrometer (500) MHz; HRESIMS were performed on a UPLC/xevo G2 Qtof spectrometer.

Preparative RP-HPLC was conducted on Agilent 1260 Infinity Series equipped with quaternary pump with Eclipse XDB-C18 (5 μm 9.4 × 250 mm) column at flow rate of 2.5 mL/min, at 210 nm UV detection using single wavelength detector. While the separation conditions were optimized on semi-preparative Agilent 1260 HPLC equipped with DAD detector by using Eclipse XDB-C18 (5 μm 4.6 × 250 mm) at flow rate of 1 mL/min. Thin layer Chromatography was performed on TLC aluminum sheets pre-coated with silica gel GF254 (EMD Chemicals, Merck KGaA, Dermstadt, Germany), visualized under UV light of 254 and 365 nm followed by 5% vanilline-H_2_SO_4_ reagent, and heat.

### Plant Material

The stems of *K. heteroclita* (Roxb.) Craib. were collected from Hupingshan mountainous region at elevation of 5,971 ft in Shimen County, Hunan, P. R. China, and identified by Prof. Wei Wang from School of Pharmacy, Hunan University of Chinese Medicine. The voucher specimen number (CEL 1280-KH) was deposited to TCM and Ethnomedicine Innovation & Development International Laboratory, School of Pharmacy, Hunan University of Chinese Medicine, Changsha, Hunan, P. R. China.

### Extraction and Isolation

Air dried plant material (100 kg) was extracted three times by using 80% ethanol in water under refluxed condition for 3 h each to produce viscous extract. This whole extract was then sequentially partitioned by liquid-liquid extraction (LLE) using non-polar, moderate to high polar organic solvents (pet-ether, chloroform, and *n*-butanol) against water to obtain wide range of metabolites.

Chloroform extract (353 g) was then subjected to silica gel column chromatography (CC) by gradient elution of solvent system PE-EtOAc (100% PE, 25% EtOAc in PE, 50% EtOAc/PE, 75% EtOAc in PE, 100% EtOAc) followed by EtOAc-MeOH elution. Subsequently the collected fractions were compiled, under the continuous guidance of TLC monitoring system to afford 12 final fractions (Kh-A to Kh-L).

Fraction Kh-C (54.7 g) was subjected to a series of silica gel column chromatography by gradient elution of PE-EtAcO v/v% to afford Kh-C-I to Kh-C-VIII. Fraction Kh-C-I yielded needle like crystals of **12** (35 mg) eluted with 10% EtAcO/PE on silica gel column. Compound **18** (9.0 mg) was isolated as white scaly crystals by silica gel CC using 4% EtAcO/PE mobile phase from Kh-C-III. Fraction Kh-C-V after successive separation afforded white solid of **13** (18.2 mg) eluted with 10% acetone, and **16** (6.0 mg) eluted with 15% acetone in PE, respectively on silica gel column. While Kh-C-VII yielded **25** (150 mg), and **24** (13 mg) by 6% and 10% EtAcO/PE, respectively, thorough silica gel CC.

Fraction Kh-D (42.5 g) after successive chromatography on silica column afforded 10 fractions. Fraction Kh-D-VI was separated on silica gel column and eluted by acetone/PE to give **14** (7.5 mg), and yielded **15** (18.0 mg) as transparent crystals by 6% acetone/CHCl_3_, furthermore, compound **8** (5.0 mg) was also separated on sephadex LH-20 CC by 1:1 MeOH/CHCl_3_ from the same sub-fraction. White crystals of **7** (4.0 g) was isolated as major compound by 15% EtAcO/PE on silica gel column.

Fraction Kh-E (25.5 g) was eluted by PE and EtAcO by gradient system. After series of separation **27** (7.6 mg) was isolated on sephadex column by 1:1 MeOH/CHCl_3_ solvent system from fraction Kh-E-V. While fraction Kh-E-VI yielded compound **19** (22.0 mg) by 50% EtAcO/DCM silica gel CC, and compound **9** (25.0 mg) was purified as feathery substance on sephadex LH-20 by 1:1 MeOH/CHCl_3_ solvent system from resulting fraction Kh-E-VI-e.

Fraction Kh-F (48.5 g) was subjected to series of silica gel CC using DCM/EtOAc followed by EtOAc/MeOH solvent system of increasing polarity. Compounds **11** (5.0 mg), and **17** (28.00 mg) were isolated on silica gel columns by 30% DCM/PE, and 20% acetone/PE, respectively, from sub-fraction. The resulting fraction Kh-F-X-e was separated on sephadex LH-20 CC using 50% MeOH in CHCl_3_ to yield sub-fraction Kh-F-X-e-2 (88 mg), which was then further purified by semi-preparative RP-HPLC. The separation conditions were optimized on analytical HPLC equipped with DAD detector. Compound **2** (20.0 mg, retention time = *t*_R_ 14.03 min), **10** (12.0 mg, retention time = *t*_R_ 17.52 min), and compound **5** (6.2 mg, retention time = *t*_R_ 20.53 min) were purified by 75% MeOH in H_2_O at flow of 2.5 mL/min using 210 nm of UV detection by using Eclipse XDB-C18 (5 μm 9.4 × 250 mm) column. While compound **28** (13.0 mg) was isolated from Kh-F-X-h eluted with 30% EtAcO/DCM on silica gel column.

Fraction Kh-G (42.8 g) was fractionated gradiently, and compound **26** (8.0 mg) was purified as precipitates during fraction collection of 15% EtAcO/DCM.

Fraction Kh-H (55.4 g) was subjected to silica gel CC by gradient elution with PE/EtOAc, and EtOAc/MeOH to yield sub fraction. Further silica gel CC was carried out for sub-fraction Kh-H-IX (6.37 g) using DCM/EtOAc and EtOAc/MeOH solvent system of increased polarity to yield fractions Kh-H-IX-a to Kh-H-IX-f. Compound **1** (10.0 mg), and compound **6** (9.5 mg) were separated on sephadex LH-20 CC eluted with 50% MeOH in CHCl_3_ from sub-fraction Kh-H-IX-c (155.78 mg), and Kh-H-IX-d-3 (85.70 mg), respectively. While fraction Kh-IX-e (2.4 g) was subjected to successive separations and ultimately compound **21** (12.5 mg, retention time = *t*_R_ 11.66 min), **22** (15.3 mg, retention time = *t*_R_ 20.10 min), **23** (10.0 mg, retention time = *t*_R_ 23.57 min), and compound **20** (6.5 mg, retention time = *t*_R_ 33.12 min) were purified by preparative HPLC by 55% MeOH//H_2_O at flow rate of 2.5 mL/min using Zorbax SB-C18 (5 μm 9.4 × 150 mm) column at 210 nm UV detection.

Finally, Fraction I (35.0 g) was chromatographed on silica gel CC eluted with a DCM/MeOH gradient system (99.5:0.5–0:100) to obtain 10 fractions. Kh-I-X (3.0 g) was subjected to silica gel CC eluted with PE/EtOAc (80:20 to 0:100) to give 12 fractions. Fraction Kh-I-X-k was purified on semi-preparative RP-HPLC, with a solvent of MeOH/H_2_O (3 mL/min, 75:25) at 225 nm, to afford compounds **3** (6.7 mg) and compounds **4** (6.7 mg).

### Spectroscopic Data

#### Xuetonglactone A (1)

Colorless prismatic crystals; ${\left[\alpha \right]}_{D}^{25}$ + 186.9 (*c* 2.44, MeOH); ECD 255 nm (Δε = + 5.64); IR ν_max_ 2,967, 1,720, 1,684, 1,599, 1,569, 1,375, 1,291, 1,246, 1,127, 1,101, 1,053, 1,028, 987, 851, 821 cm^−1^; ^1^H-NMR (500 MHz, CDCl_3_) and ^13^C-NMR (125 MHz, CDCl_3_) data, see Tables 1, 2, respectively; (+)-HRESIMS *m/z* 559.2671 [M + Na]^+^ (calcd for C_32_H_40_O_7_ + Na, 559.2672).

#### Xuetonglactone B (2)

White amorphous; ${\left[\alpha \right]}_{D}^{25}\;+$ 242.7 (*c* 3.94, MeOH); ECD 255 nm (Δε = + 5.80); IR ν_max_ 3,449, 2,952, 1,720, 1,686, 1,669, 1,597, 1,567, 1,373, 1,289, 1,248, 1,127, 1,049, 1,026, 987, 853, 821 cm^−1^; ^1^H-NMR (500 MHz, CDCl_3_) and ^13^C-NMR (125 MHz, CDCl_3_) data, see Tables 1, 2, respectively; (+)-HRESIMS *m/z* 561.2828 [M + Na]^+^ (calcd for C_32_H_42_NaO_7_ + Na, 561.2829), and (–)-HRESIMS *m/z* 573.2612 (calcd for C_32_H_42_O_7_ + Cl, 537.2619).

#### Xuetonglactone C (3)

White amorphous solid; ${\left[\alpha \right]}_{D}^{25}$ 108.1 (*c* 2.44, MeOH); ECD 273 nm (Δε = + 0.41); IR ν_max_ 3,446, 2,929, 1,724, 1,687, 1,656, 1,375, 1,291, 1,249, 1,131, 1,024, 990, 825 cm^−1^; ^1^H-NMR (600 MHz, CDCl_3_) and ^13^C-NMR (150 MHz, CDCl_3_) data, see Tables 1, 2, respectively; (+)-HRESIMS *m/z* 561.2836 [M + Na]^+^ (calcd for C_32_H_42_O_7_ + Na, 561.2828).

#### Xuetonglactone D (4)

White amorphous; ${\left[\alpha \right]}_{D}^{25}$ 41.8 (*c* 1.67, MeOH); ECD 258 nm (Δε = + 5.53); IR ν_max_ 3,449, 2,988, 2,923, 2,850, 1,724, 1,465, 1,381,1,243, 1,125, 1,032, 988 cm^−1^; ^1^H-NMR (600 MHz, CDCl_3_) and ^13^C-NMR (150 MHz, CDCl_3_) data, see Tables 1, 2, respectively; (+)-HRESIMS *m/z* 577.2772 [M + Na]^+^ (calcd for C_32_H_42_O_8_ + Na, 577.2777).

#### Xuetonglactone E (5)

White prismatic crystals; mp 235.1–236.6°C (MeOH); ${\left[\alpha \right]}_{D}^{25}$ −8.8 (*c* 0.1, MeOH); ECD 272 nm (Δε = + 3.31); IR ν_max_ 2,946, 1,721, 1,679, 1,558, 1,457, 1,381, 1,243, 1,106, 1,035, 914 cm^−1^; ^1^H-NMR (600 MHz, CDCl_3_) and ^13^C-NMR (150 MHz, CDCl_3_) data, see Tables 1, 2, respectively; (+)-HRESIMS *m/z* 547.3047 [M + Na]^+^ (calcd for C_32_H_44_O_6_ + Na, 547.3036).

#### Xuetonglactone F (6)

White amorphous; ${\left[\alpha \right]}_{D}^{25}$ + 66.7 (*c* 1.56, MeOH); ECD 266 nm (Δε = + 2.41); IR ν_max_ 2,916, 2,849, 1,736, 1,718, 1,684, 1,459, 1,377, 1,289, 1,239, 1,120, 993, 915, 825 cm^−1^; ^1^H-NMR (500 MHz, CDCl_3_) and ^13^C-NMR (125 MHz, CDCl_3_) data, see Tables 1, 2, respectively; (–)-HRESIMS *m/z* 617.2884 [M + Cl]^−^ (calcd for C_34_H_46_O_8_ + Cl, 617.2881).

#### X-Ray Crystallographic Analysis

X-ray crystallographic data of **1, 5**, and **7** were obtained using a Bruker APEX-II CCD diffractometer with Cu *K* radiation, = 1.54178 Å The CCDC numbers for **1, 5**, and **7** contain the supplementary crystallographic data, which can be obtained free of charge via http://www.ccdc.cam.ac.uk/conts/retrieving.html.

#### Crystal Data for Xuetonglactone A (1)

C_32_H_40_O_7_, M = 536.64, colorless crystals, Orthorhombic, a = 7.1986 (3) Å, b = 13.9377 (6) Å, c = 28.5609 (13) Å, α = 90.00°, β = 90.00°, γ = 90.00°, *V* = 2865.6 (2) Å^3^, s *P*2_1_2_1_2_1_, *T* = 296 K, Z = 4, μ(Cu Kα) = 0.70 mm^−1^, 22,617 reflections measured, 5,112 independent reflections (*R*_int_ = 0.073). Final R indices [*I* >2σ(*I*)]: R1 = 0.052, wR2 = 0.164. Flack parameter: −0.10 (13). CCDC number: 1859825.

#### Crystal Data for Xuetonglactone E (5)

C_32_H_44_O_6_·H_2_O, M = 542.69, colorless crystal, Orthorhombic, a = 10.9410 (7) Å, b = 14.5893 (9) Å, c = 18.2354 (11) Å, α = 90.00°, β = 90.00°, γ = 90.00°, *V* = 2910.8 (3) Å^3^, space group *P*2_1_2_1_2_1_, T = 296 K, Z = 4, μ(Cu Kα) = 0.69 mm^−1^, 31,389 reflections measured, 5,410 independent reflections (Rint = 0.040). Final R indices [*I* > 2σ(*I*)]: R1 = 0.036, wR2 = 0.109. Flack parameter: 0.06 (4). CCDC number: 1859823.

#### Crystal Data for Xuetongsu (7)

4(C_30_H_44_O_4_)·O, M = 1,890.60, colorless crystal, Monoclinic, a = 46.638 (2) Å, b = 7.4805 (4) Å, c = 7.8525 (4) Å, α = 90.00°, β = 91.597(2)°, γ = 90.00°, *V* = 2738.5 (2) Å^3^, space group C2_,_ T = 296.15 K, Z = 1, μ(Cu Kα) = 0.59 mm^−1^, 10,267 reflections measured, 3,797 independent reflections (Rint = 0.098). Final R indices [*I* > 2s(*I*)]: R1 = 0.040, wR2 = 0.140. Flack parameter: 0.09 (13). CCDC number: 1859822.

### Biological Activity Evaluation

#### Inhibition of iNOS Activity

The assay was performed in mouse macrophages (RAW264.7) cultured in phenol red-free RPMI medium with 10% bovine calf serum, 100 U/mL penicillin G sodium, and 100 μg/mL streptomycin. The cells were seeded in 96-well plates at the density of 1 × 10^5^ cells/well, and incubated for 24 h for a confluency of 75% or more. The cells were treated with the test compounds, and after 30 min of incubation, lipopolysaccharide (LPS, Sigma-Aldrich, St. Louis, MO, USA) (5 μg/mL) was added and further incubated for 24 h. The activity of iNOS was determined in terms of the concentration of NO by measuring the level of nitrite in the cell culture supernatant using Griess reagent (Sigma-Aldrich, St. Louis, MO, USA). Percent inhibition of nitrite production by the test compound was calculated in comparison to the vehicle control. IC_50_ values were obtained from dose response curves. Parthenolide was used as the positive control (Zhao et al., 2014).

#### Cytotoxicity Assay

Cell viability was determined by a MTT assay (Roche Diagnosis, Indianapolis, IN). Briefly, BGC-823 and HeLa cell lines were seeded at 6 × 10^3^ cells/well in 96-well plates. Cells were allowed to adhere for overnight, and then the cells were changed to fresh medium containing various concentrations natural compound dissolved in DMSO. After 48 h incubation, the growth of cells was measured. The effect on cell viability was assessed as the percent cell viability compared with untreated control group, which were arbitrarily assigned 100% viability. The compound concentration required to cause 50% cell growth inhibition (IC_50_) was determined by interpolation from dose–response curves. All experiments were performed in triplicate, and paclitaxel was used as the positive control (Hayon et al., 2003).

## Conclusions

To sum up, four new highly oxygenated lanostane-type triterpenoids xuetonglactones A–D (**1**–**4**) and two highly oxygenated cycloartane-type triterpenoids xuetonglactones E–F (**5**–**6**), along with 22 known compounds (**7**–**28**) were isolated from stems of *K. heteroclita*. To the best of our knowledge xuetonglactones A (**1**) endowed with unprecedented 20,21-α-epoxide functionality, and xuetonglactones D (**4**) possessed rare 19-α hydroperoxyl moiety, their absolute configurations were determined by X-ray diffraction and ECD data analysis. Moreover, bioassays indicated that **1** and **2** showed inhibition of iNOS activity in LPS-induced macrophages, **6**, **7**, **8**, and **24** showed potent cytotoxicities against HeLa and BGC 823 cancer cell lines. Notably, this study has further enriched the chemical diversity of highly oxygenated triterpenoidal skeletons, which might trigger research rigor among synthetic and medicinal chemistry community.

## Data Availability Statement

The raw data supporting the conclusions of this article will be made available by the authors, without undue reservation, to any qualified researcher.

## Author Contributions

WW and DL conceived and designed the idea of the study. NS performed the isolation work. BL performed physical data analysis. NS and BL prepared the first draft of the manuscript. LC helped in collection of literature and assisting in crystallization. JZ performed the NMR data acquisition. YJ and AW contributed in analysis of NMR data. MD performed the bioassays of the compounds. MC and AR contributed in revision and final data analyses. IK provided the core facility to acquire NMR and other spectroscopic data. All authors read and approved the final manuscript.

### Conflict of Interest

The authors declare that the research was conducted in the absence of any commercial or financial relationships that could be construed as a potential conflict of interest.
