# Supplementary material for: Xuetonglactones A–F: Highly Oxidized Lanostane and Cycloartane Triterpenoids From Kadsura heteroclita Roxb. Craib
Source: Front Chem. 2020 Jan 21;7:935. doi: 10.3389/fchem.2019.00935 (PMC6990113; doi:10.3389/fchem.2019.00935)
Supplement: Supplementary file 1 [file Data_Sheet_1.pdf]

Supporting information

Xuetonglactones A-F: Highly Oxidized Lanostane and Cycloartane Triterpenoids from *Kadsura heteroclita* Roxb. Craib.

Nuzhat Shehla[2,1†], Bin Li[1,2†], Liang Cao[1], Jianping Zhao[4], Yuqing Jian[1], Muhammad Daniyal[1], Atia-tul-Wahab[3], Ikhlas A. Khan[4], Duan-fang Liao[1], Atta-ur-Rahman[2], M. Iqbal Choudhary[2,1,3]*, and Wei Wang[1,2]*

[1] TCM and Ethnomedicine Innovation & Development International Laboratory, Academician Atta-ur-Rahman Belt and Road Traditional Medicine Research Center, School of Pharmacy, Hunan University of Chinese Medicine, Changsha, Hunan, People's Republic of China.

[2] H. E. J. Research Institute of Chemistry, International Center for Chemical and Biological Sciences, University of Karachi, Karachi, Pakistan.

[3] Dr. Panjwani Center for Molecular Medicine and Drug Research, International Center for Chemical and Biological Sciences, University of Karachi, Karachi, Pakistan.

[4] National Center for Natural Products Research, Research Institute of Pharmaceutical Sciences, University of Mississippi, MS, United States.

* Corresponding authors:

Tel: +86-136-5743-8606; Fax: +86-0731-8845-8227; Email: wangwei402@hotmail.com

Tel: +92-21-4824924, 4819010; Fax: +92-21-481901; E-mail: iqbal.choudhary@iccs.edu

[†] These authors contributed equally to this work.

Electronic Supplementary Information (ESI) available: The 1D- and 2D-NMR spectra and HRESIMS of compounds **1**–**6** (PDF).

Contents of supporting information

Figure S1. ^{1}H NMR (500 MHz, $CDCl_3$) spectrum of compound **1**-----------------------------4
Figure S2.^{13}C NMR (125 MHz, $CDCl_3$) spectrum of compound **1**-----------------------------4
Figure S3. ^{1}H-^{1}H COSY spectrum of compound **1**--5
Figure S4. HSQC spectrum of compound **1**---5
Figure S5. HMBC spectrum of compound **1**---6
Figure S6. ROESY spectrum of compound **1**--6
Figure S7. CD spectrum of compound **1**--7
Figure S8. HRESI-MS spectrum of compound **1**---7
Figure S9. ^{1}H NMR (500 MHz, $CDCl_3$) spectrum of compound **2**----------------------------8
Figure S10.^{13}C NMR (125 MHz, $CDCl_3$) spectrum of compound **2**----------------------------8
Figure S11. ^{1}H-^{1}H COSY spectrum of compound **2**--9
Figure S12. HSQC spectrum of compound **2**---9
Figure S13. HMBC spectrum of compound **2**---10
Figure S14. ROESY spectrum of compound **2**--10
Figure S15. CD spectrum of compound **2**---11
Figure S16. HRESI-MS spectrum of compound **2**---11
Figure S17. ^{1}H NMR (600 MHz, $CDCl_3$) spectrum of compound **3**-----------------------------12
Figure S18.^{13}C NMR (150 MHz, $CDCl_3$) spectrum of compound **3**-----------------------------12
Figure S19. ^{1}H-^{1}H COSY spectrum of compound **3**---13
Figure S20. HSQC spectrum of compound **3**---13
Figure S21. HMBC spectrum of compound **3**---14
Figure S22. NOESY spectrum of compound **3**---14
Figure S23. CD spectrum of compound **3**---15
Figure S24. HRESI-MS spectrum of compound **3**--15
Figure S25. ^{1}H NMR (600 MHz, $CDCl_3$) spectrum of compound **4**-----------------------------16
Figure S26.^{13}C NMR (150 MHz, $CDCl_3$) spectrum of compound **4**-----------------------------16
Figure S27. ^{1}H-^{1}H COSY spectrum of compound **4**---17
Figure S28. HSQC spectrum of compound **4**---17
Figure S29. HMBC spectrum of compound **4**---18
Figure S30. NOESY spectrum of compound **4**---18
Figure S31. CD spectrum of compound **4**---19
Figure S32. HRESI-MS spectrum of compound **4**---19
Figure S33. ^{1}H NMR (600 MHz, $CDCl_3$) spectrum of compound **5**-----------------------------20
Figure S34.^{13}C NMR (150 MHz, $CDCl_3$) spectrum of compound **5**-----------------------------20
Figure S35. ^{1}H-^{1}H COSY spectrum of compound **5**---21
Figure S36. HSQC spectrum of compound **5**---21
Figure S37. HMBC spectrum of compound **5**---22
Figure S38. ROESY spectrum of compound **5**--22
Figure S39. CD spectrum of compound **5**---23
Figure S40. HRESI-MS spectrum of compound **5**---23
Figure S41. ^{1}H NMR (500 MHz, $CDCl_3$) spectrum of compound **6**-----------------------------24
Figure S42.13C NMR (125 MHz, CDCl3) spectrum of compound **6**-----------------------------24
Figure S43. ^{1}H-^{1}H COSY spectrum of compound **6**---25

Figure S44. HSQC spectrum of compound **6**--25
Figure S45. HMBC spectrum of compound **6**--26
Figure S46. ROESY spectrum of compound **6**---26
Figure S47. CD spectrum of compound **6**--27
Figure S48. HRESI-MS spectrum of compound **6**--27

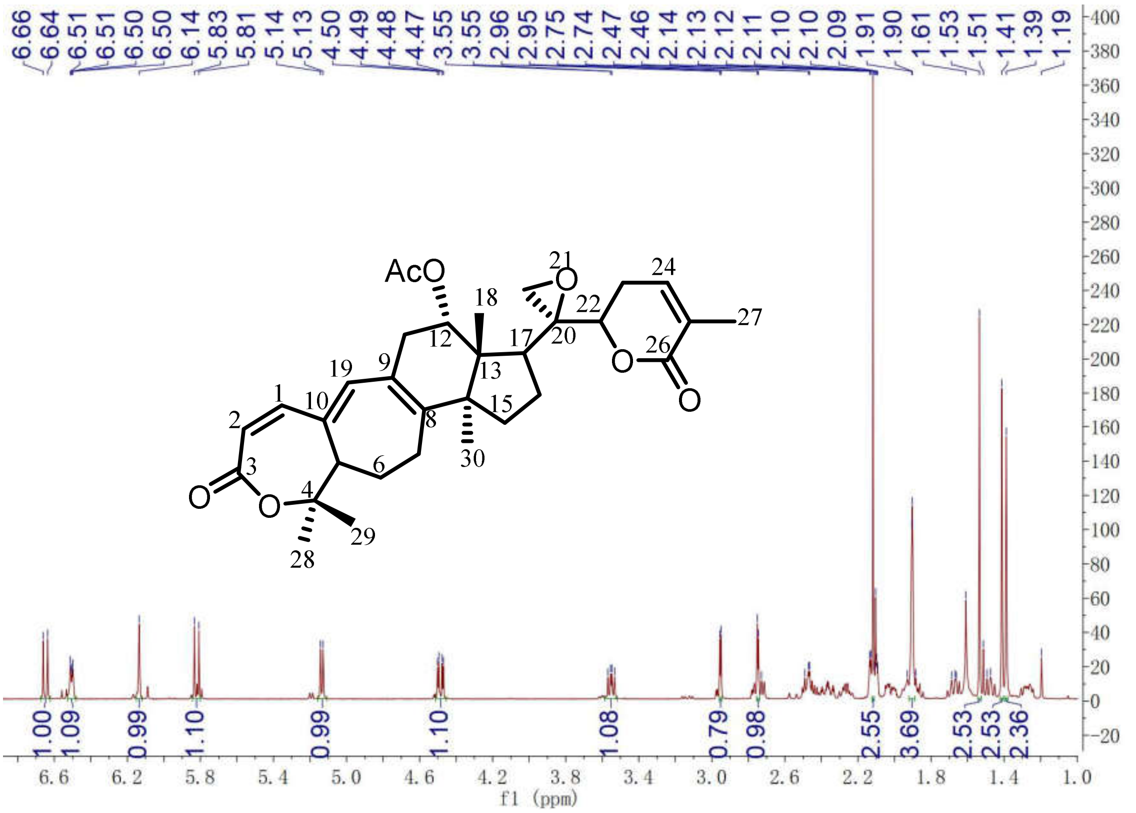

Figure S1. ^{1}H NMR (500 MHz, $CDCl_3$) spectrum of compound **1**

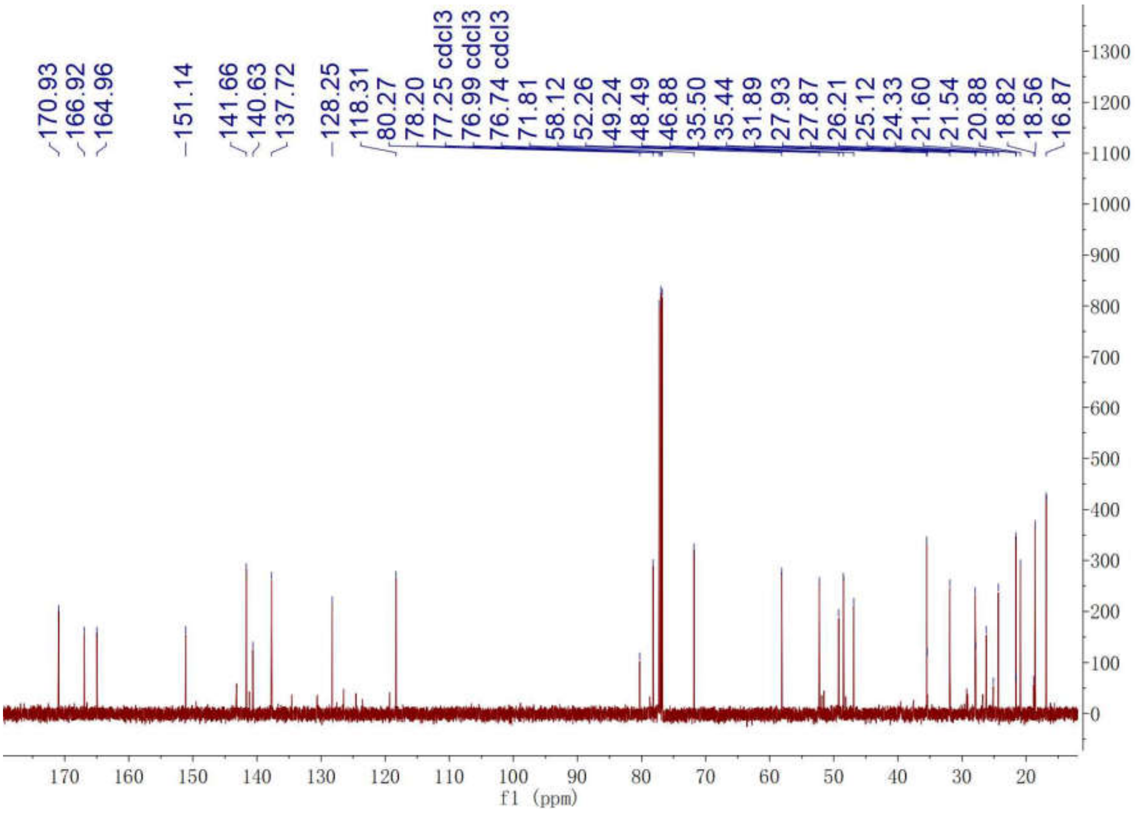

Figure S2.^{13}C NMR (125 MHz, $CDCl_3$) spectrum of compound **1**

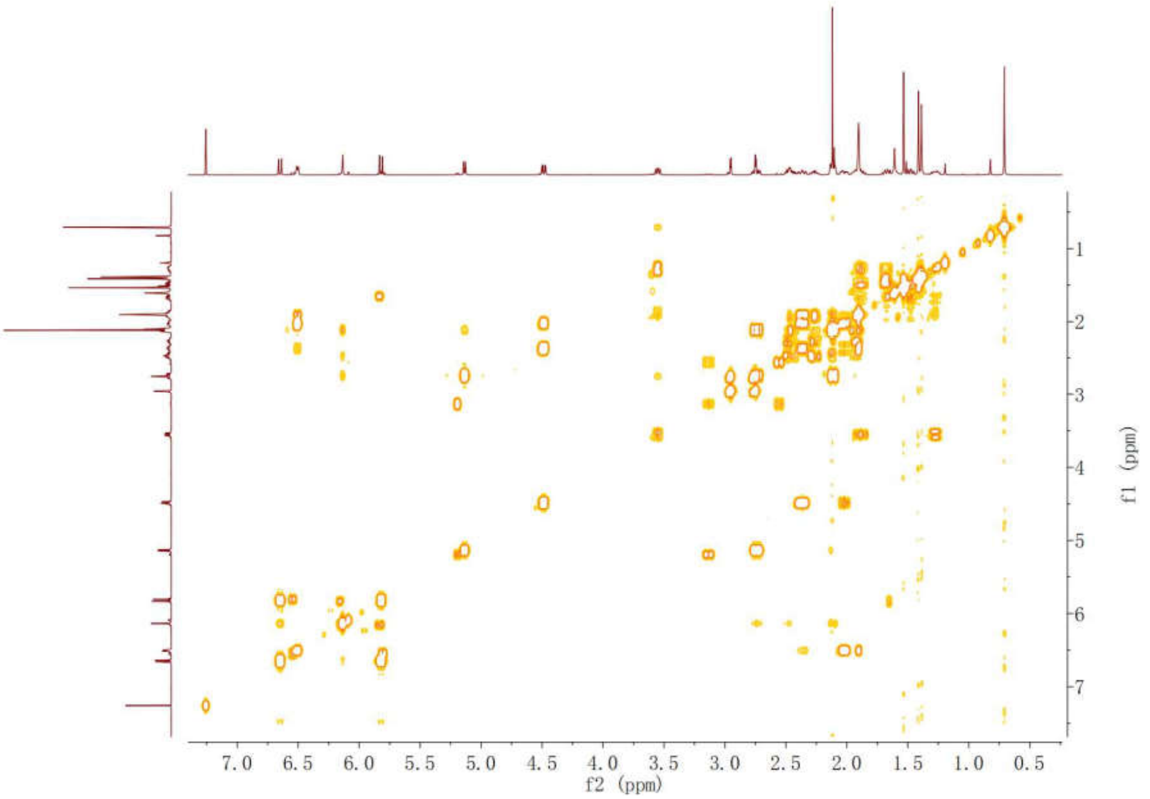

Figure S3. ^{1}H-^{1}H COSY spectrum of compound **1**

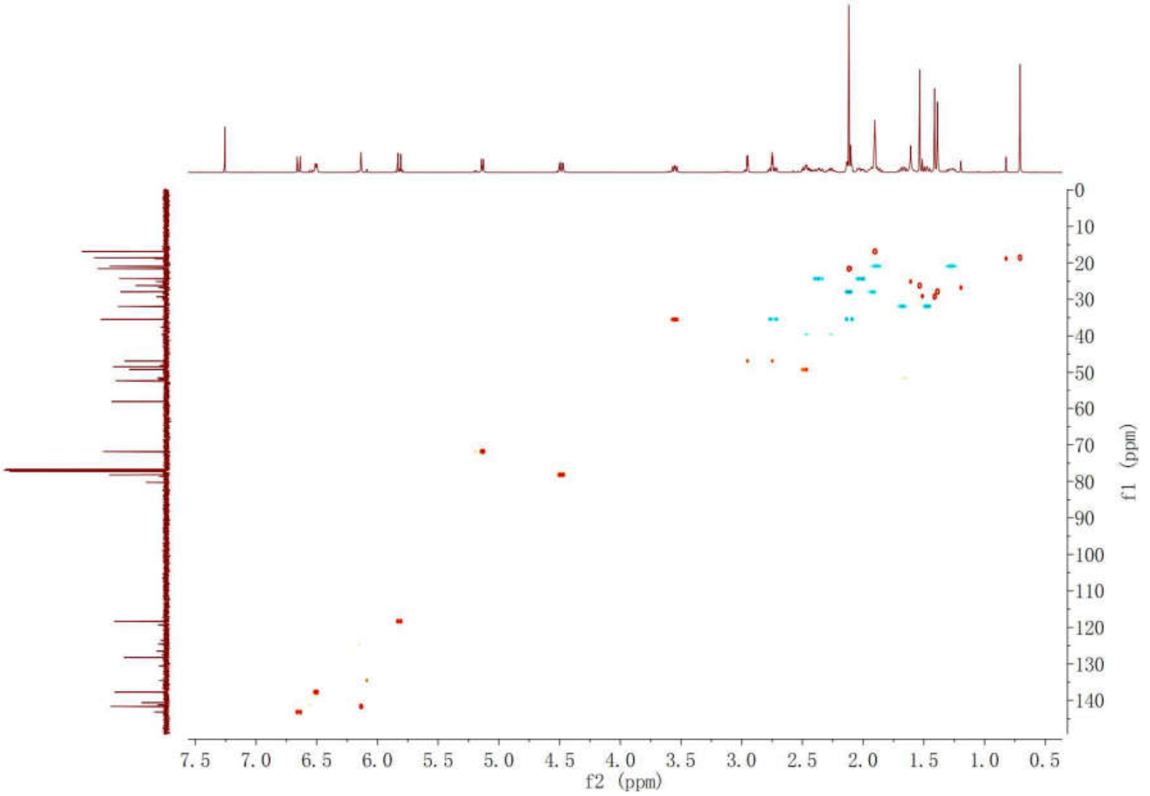

Figure S4. HSQC spectrum of compound **1**

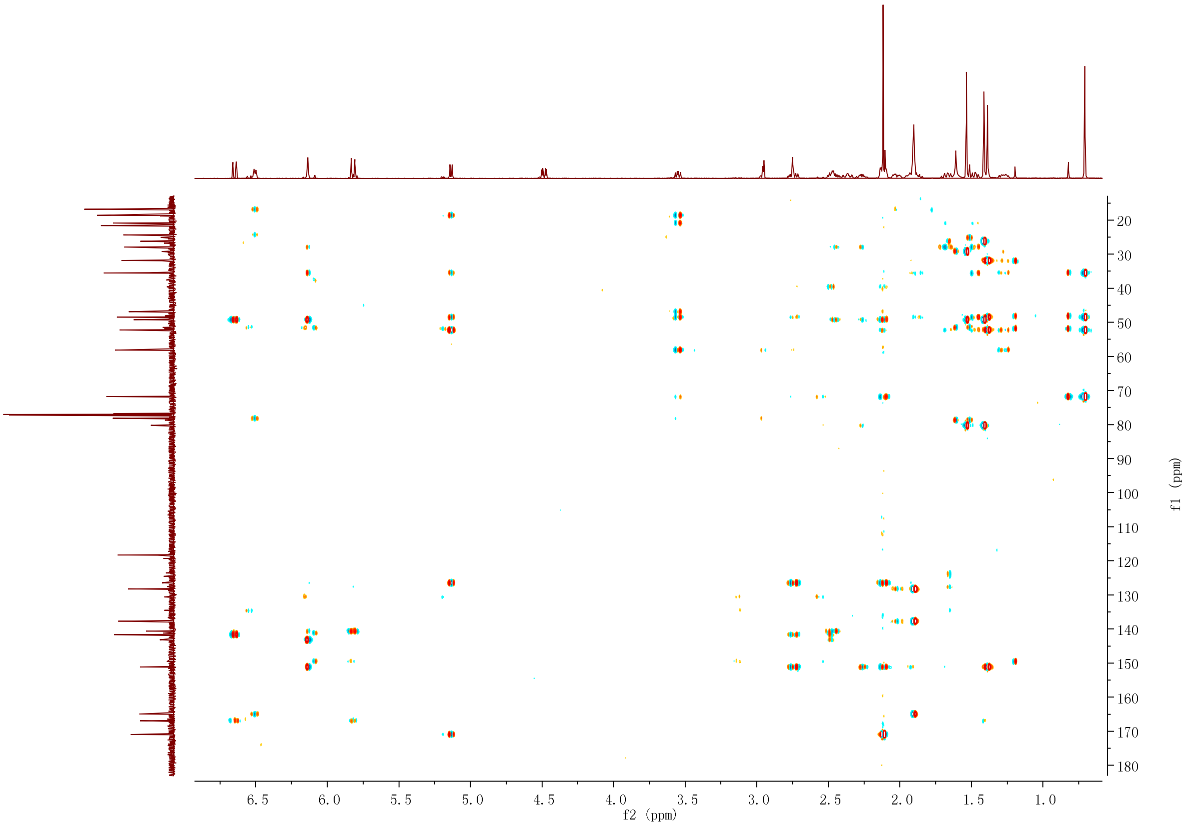

Figure S5. HMBC spectrum of compound **1**

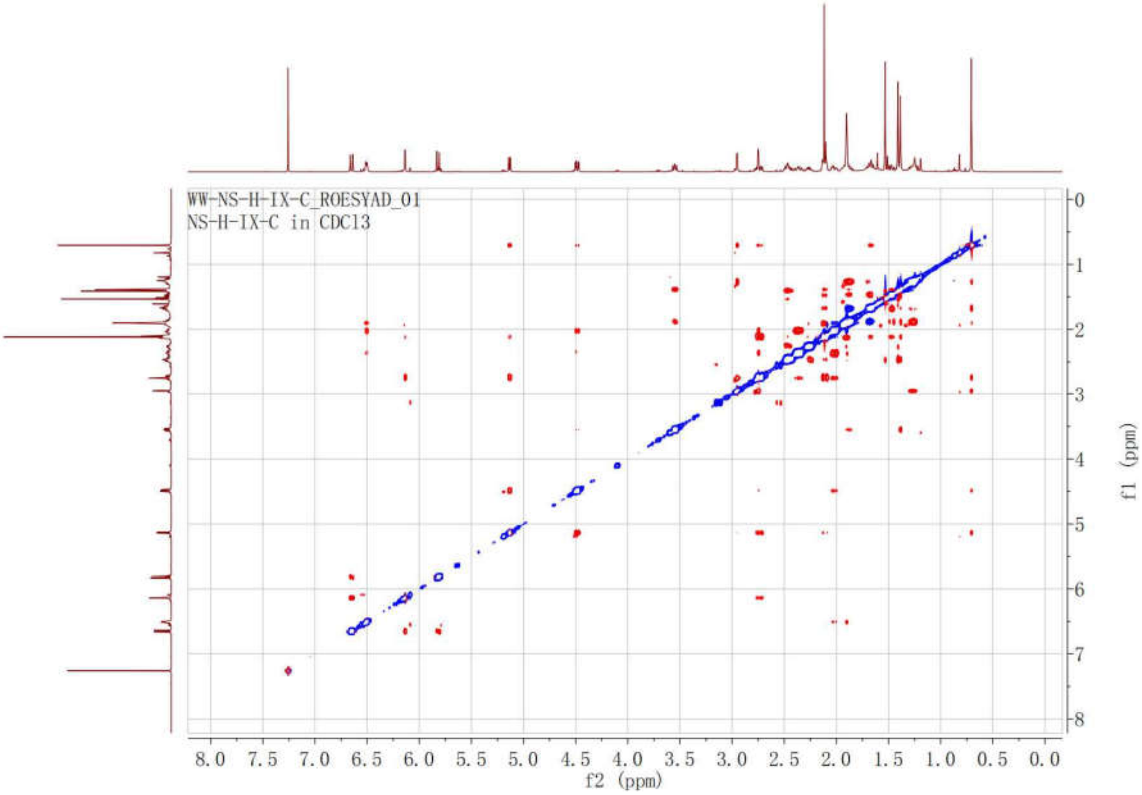

Figure S6. ROESY spectrum of compound **1**

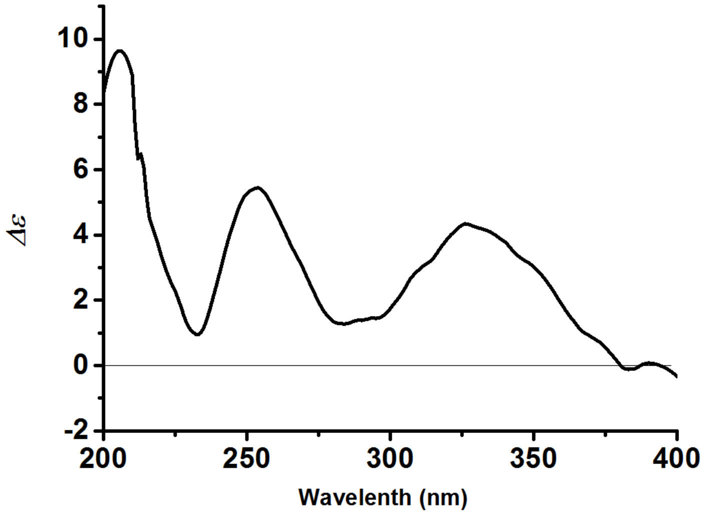

Figure S7. CD spectrum of compound **1**

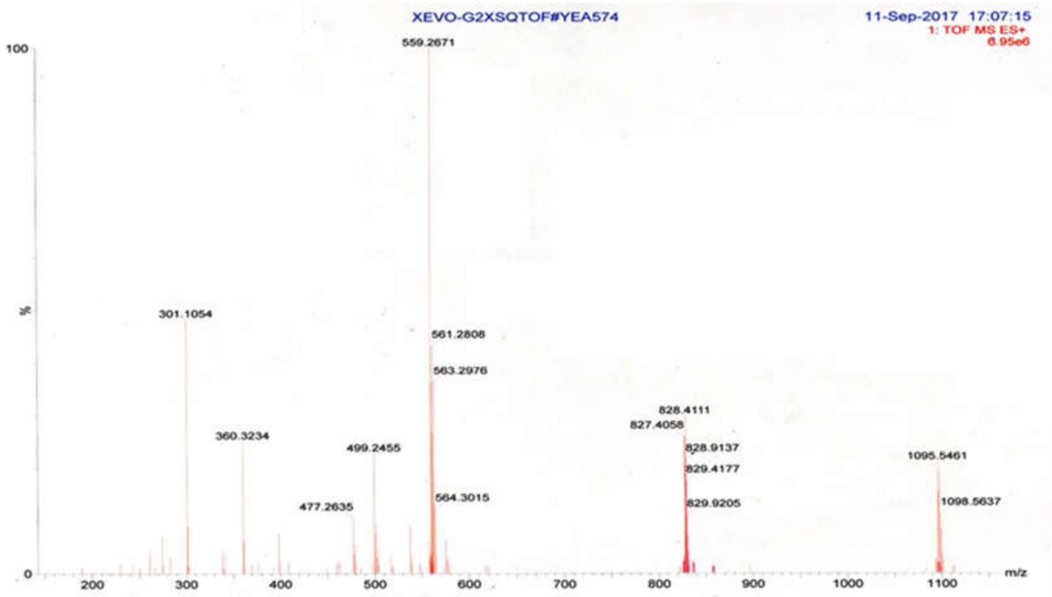

Figure S8. HRESI-MS spectrum of compound **1**

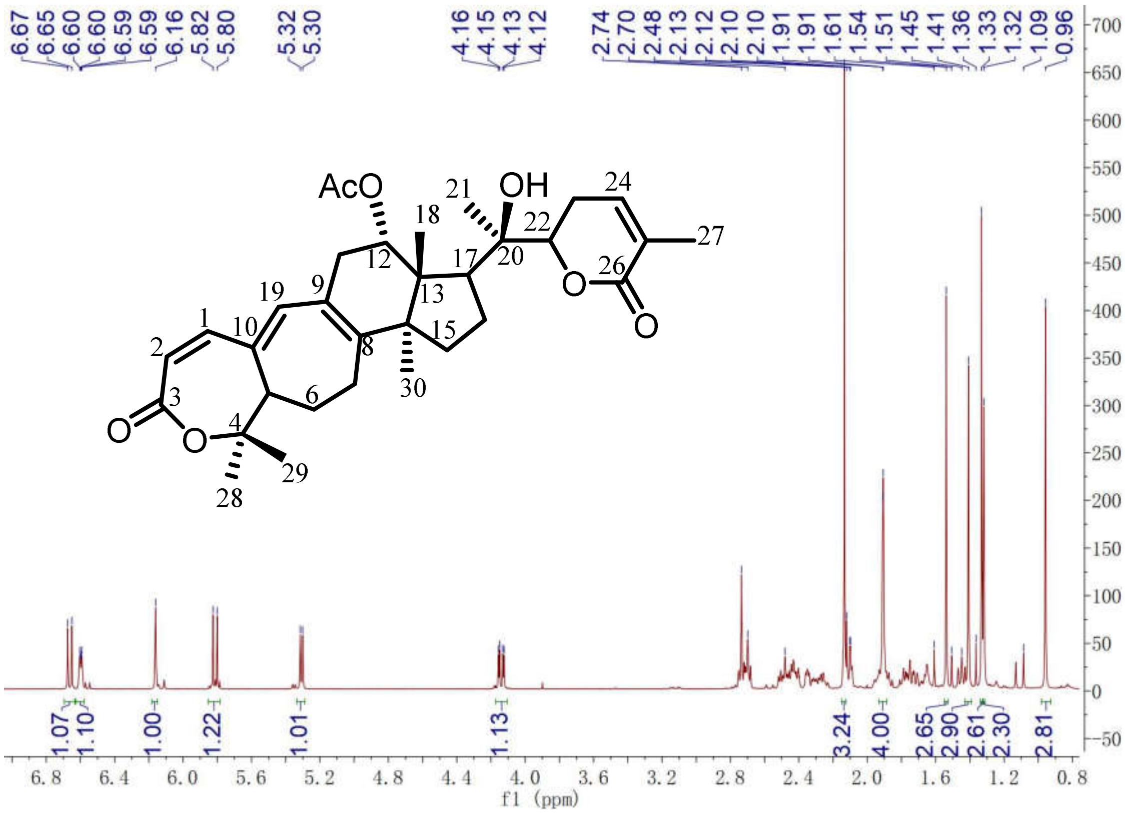

Figure S9. ^{1}H NMR (500 MHz, $CDCl_3$) spectrum of compound **2**

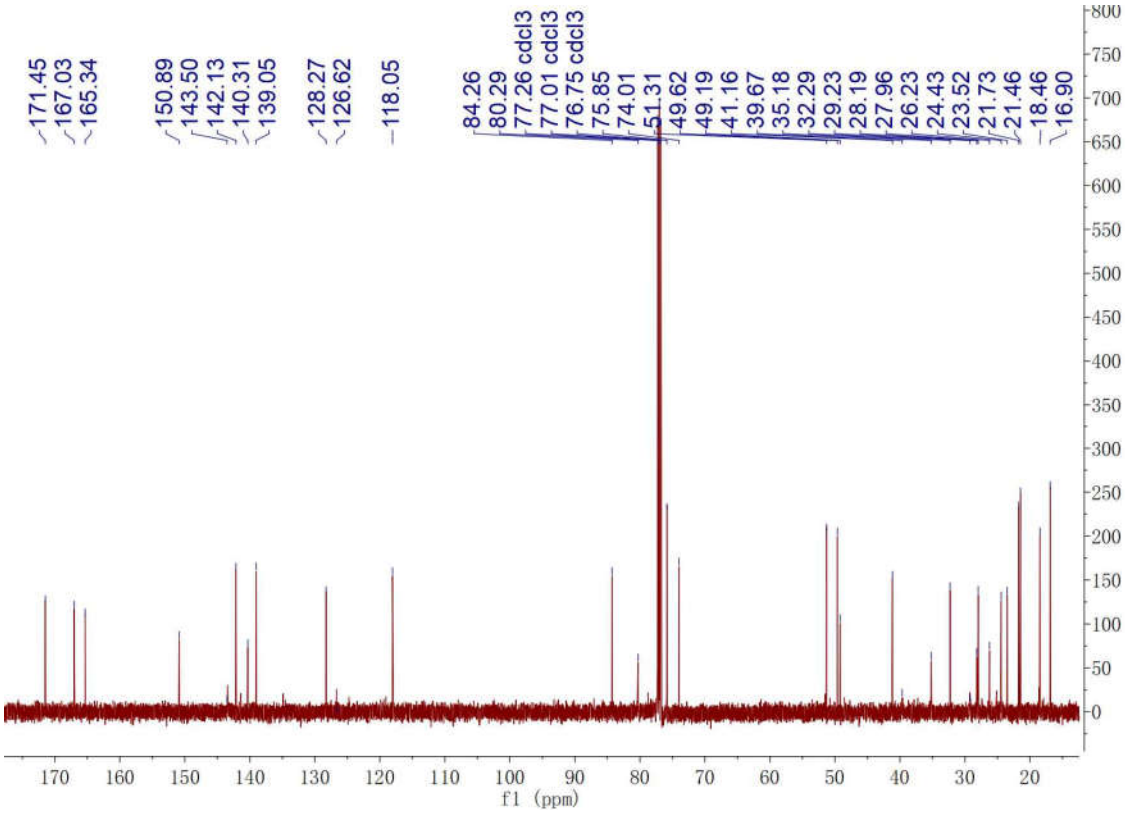

Figure S10. ^{13}C NMR (125 MHz, $CDCl_3$) spectrum of compound **2**

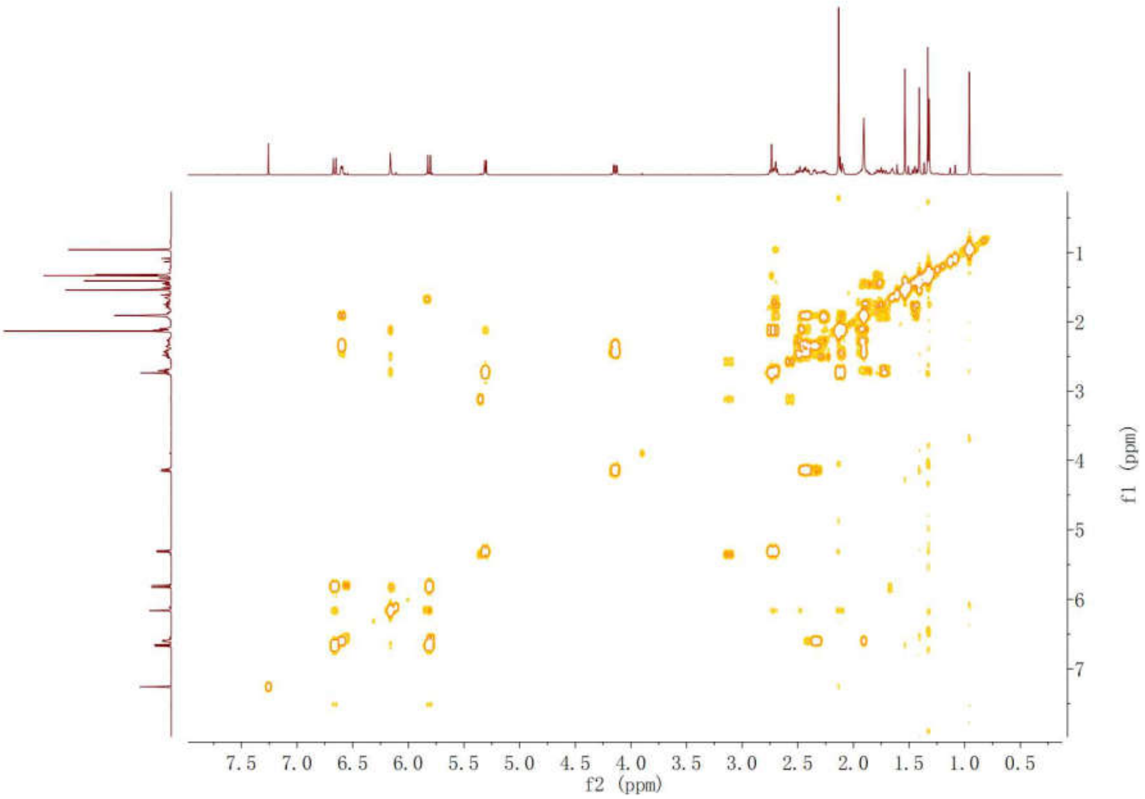

Figure S11. ^{1}H-^{1}H COSY spectrum of compound **2**

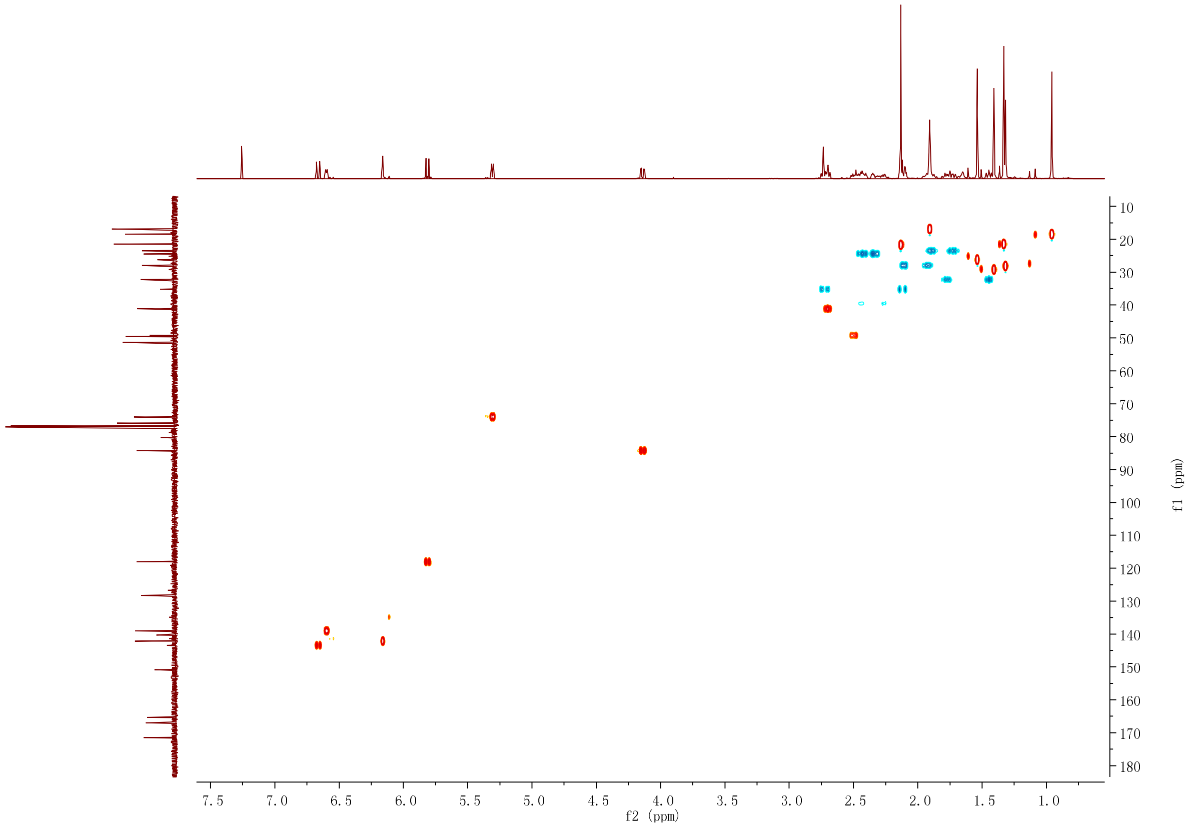

Figure S12. HSQC spectrum of compound **2**

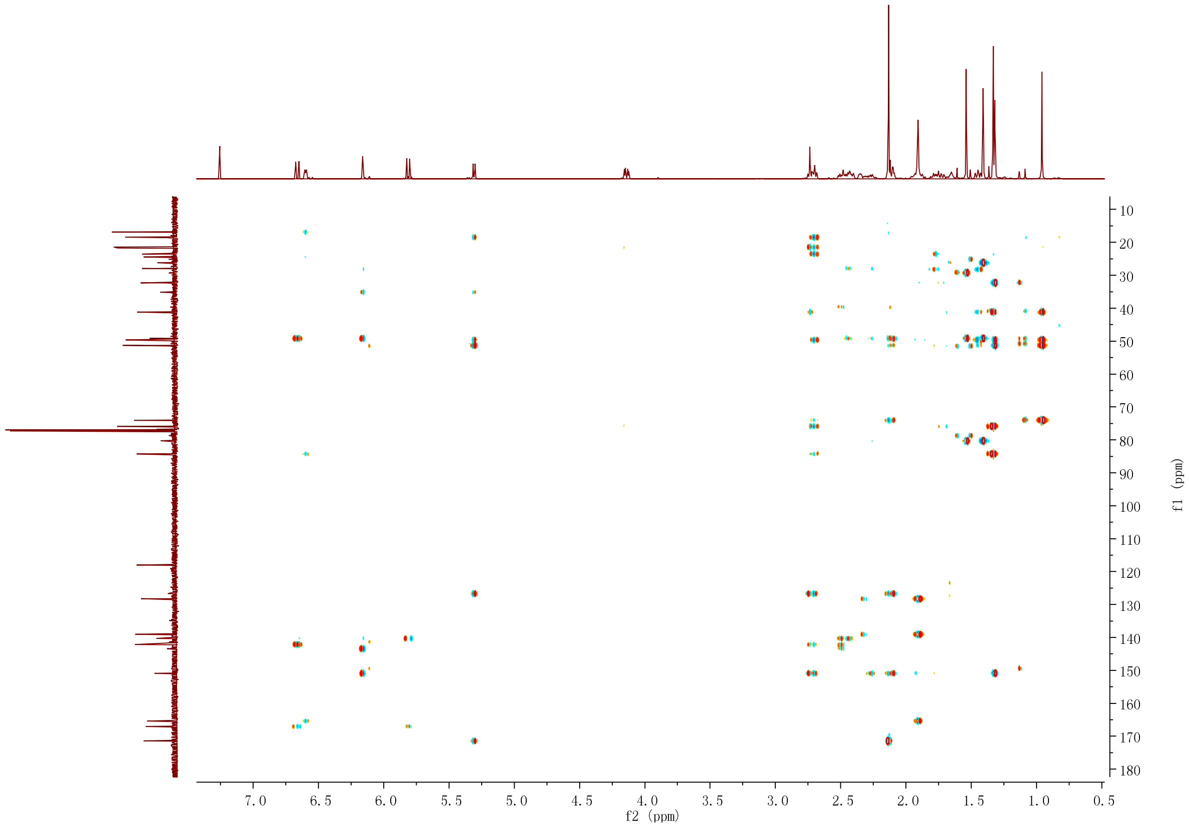

Figure S13. HMBC spectrum of compound **2**

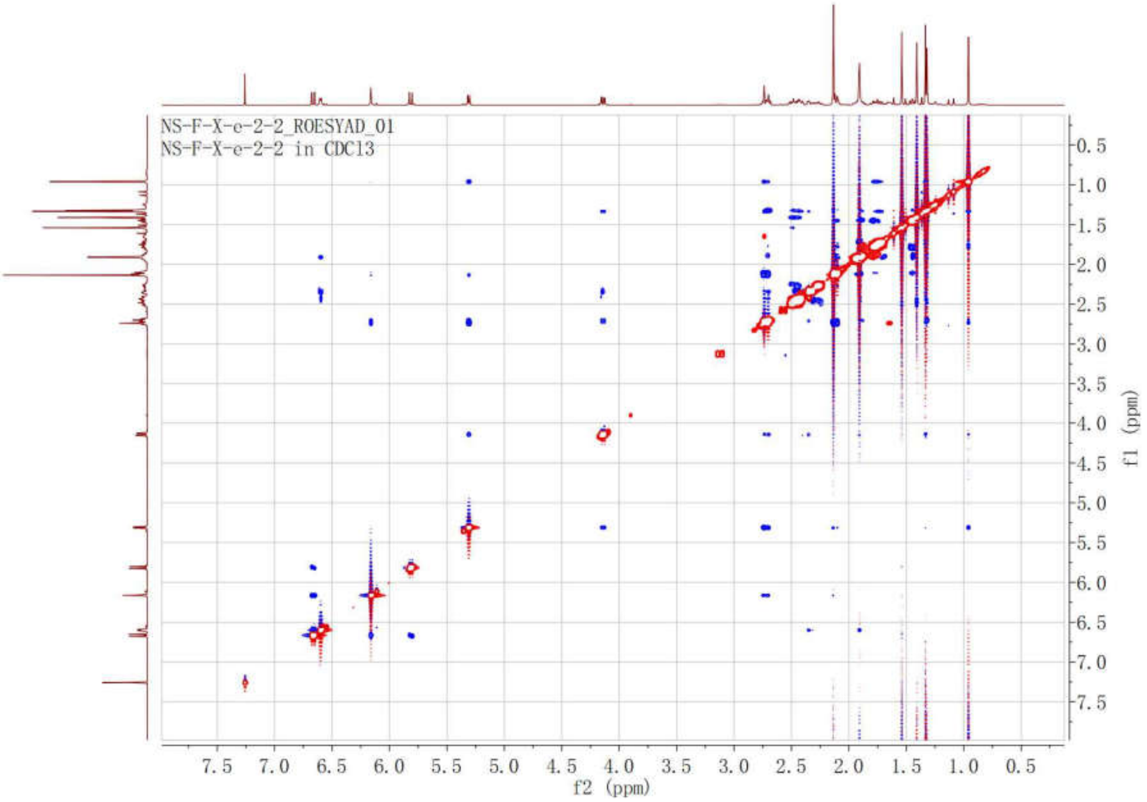

Figure S14. ROESY spectrum of compound **2**

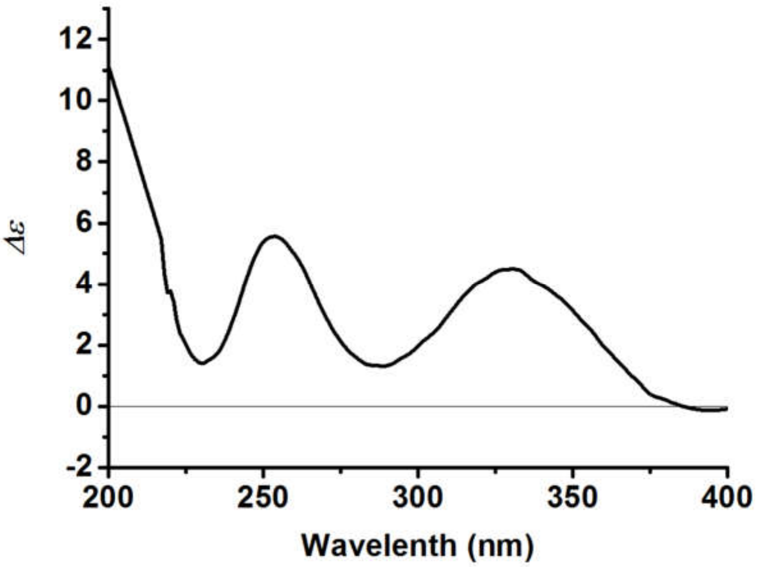

Figure S15. CD spectrum of compound **2**

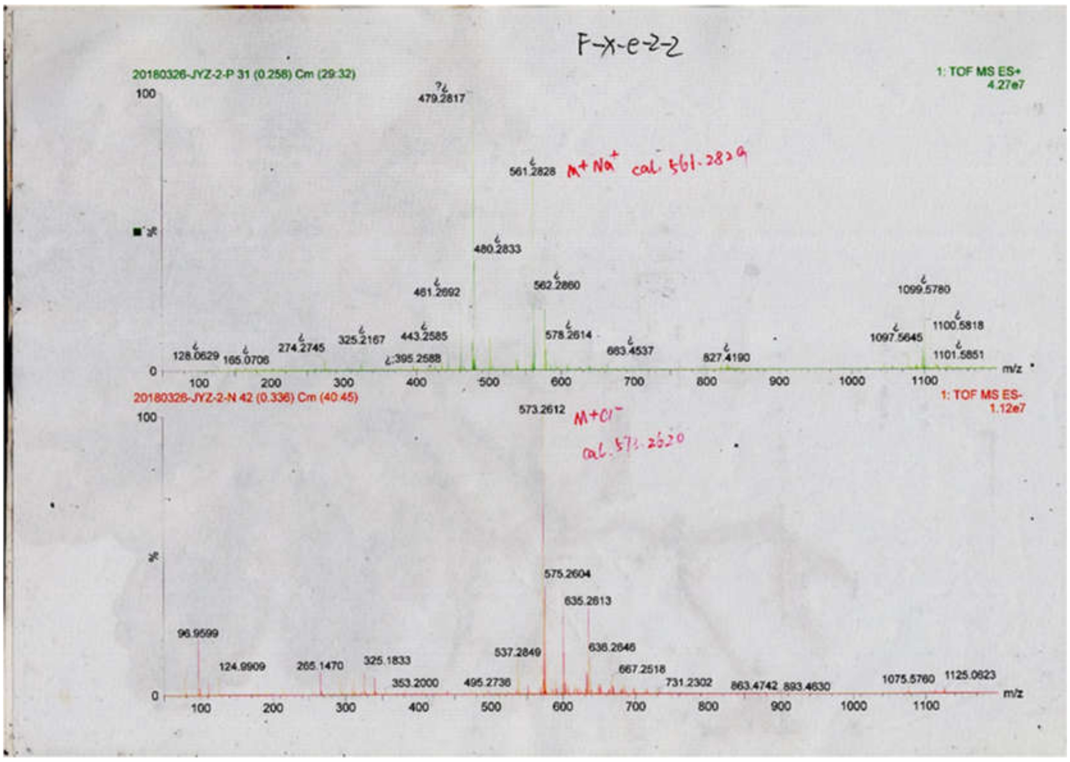

Figure S16. HRESI-MS spectrum of compound **2**

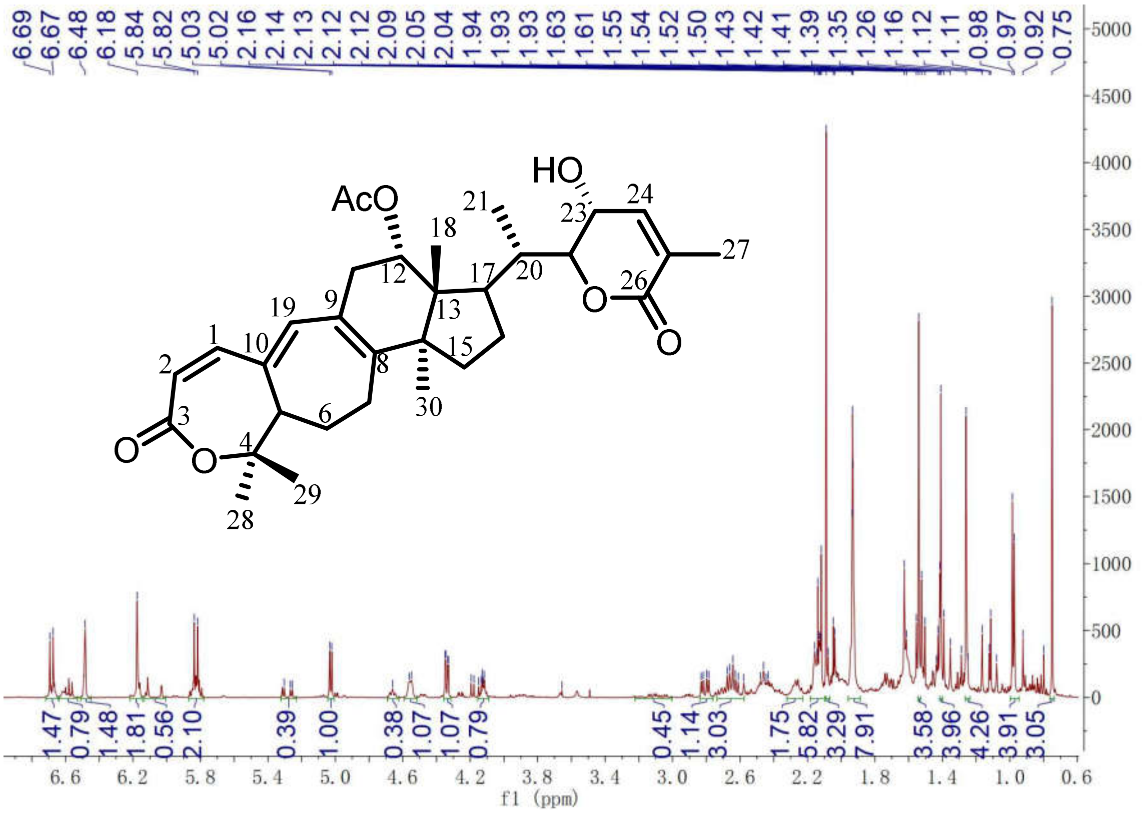

Figure S17. ^{1}H NMR (600 MHz, $CDCl_3$) spectrum of compound **3**

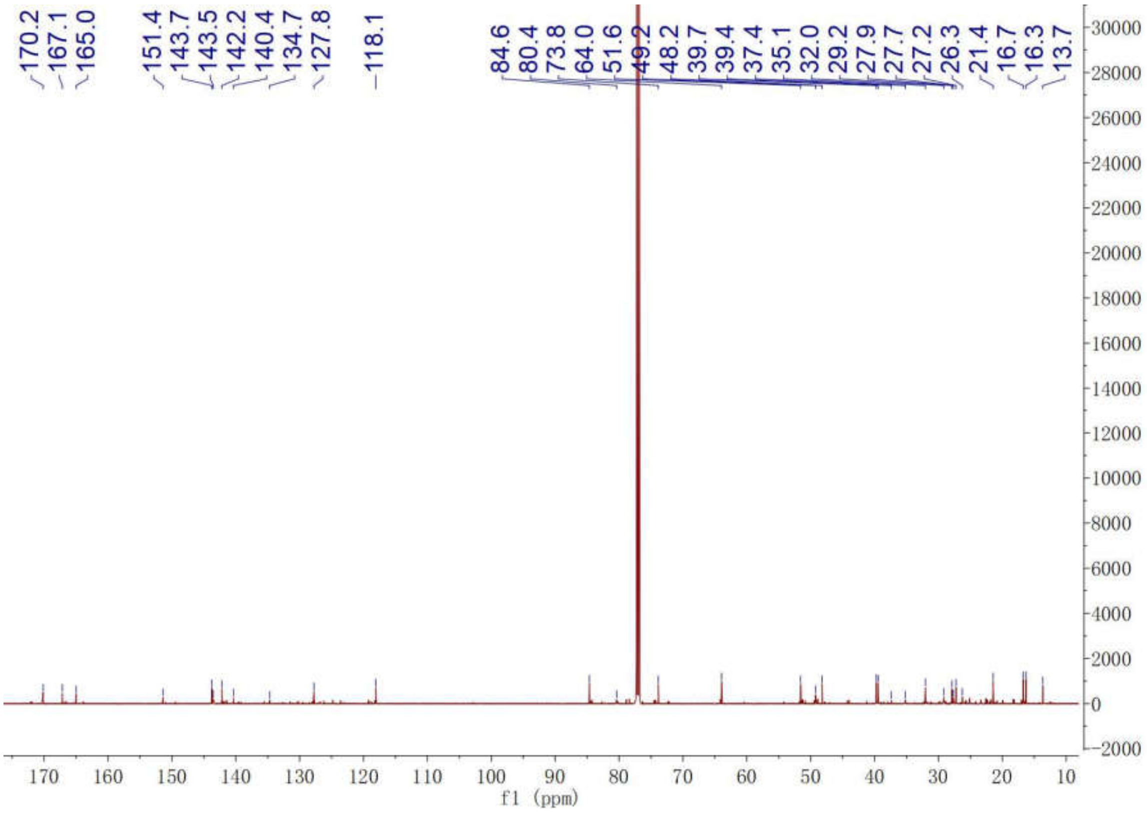

Figure S18. [13] C NMR (150 MHz, $CDCl_3$) spectrum of compound **3**

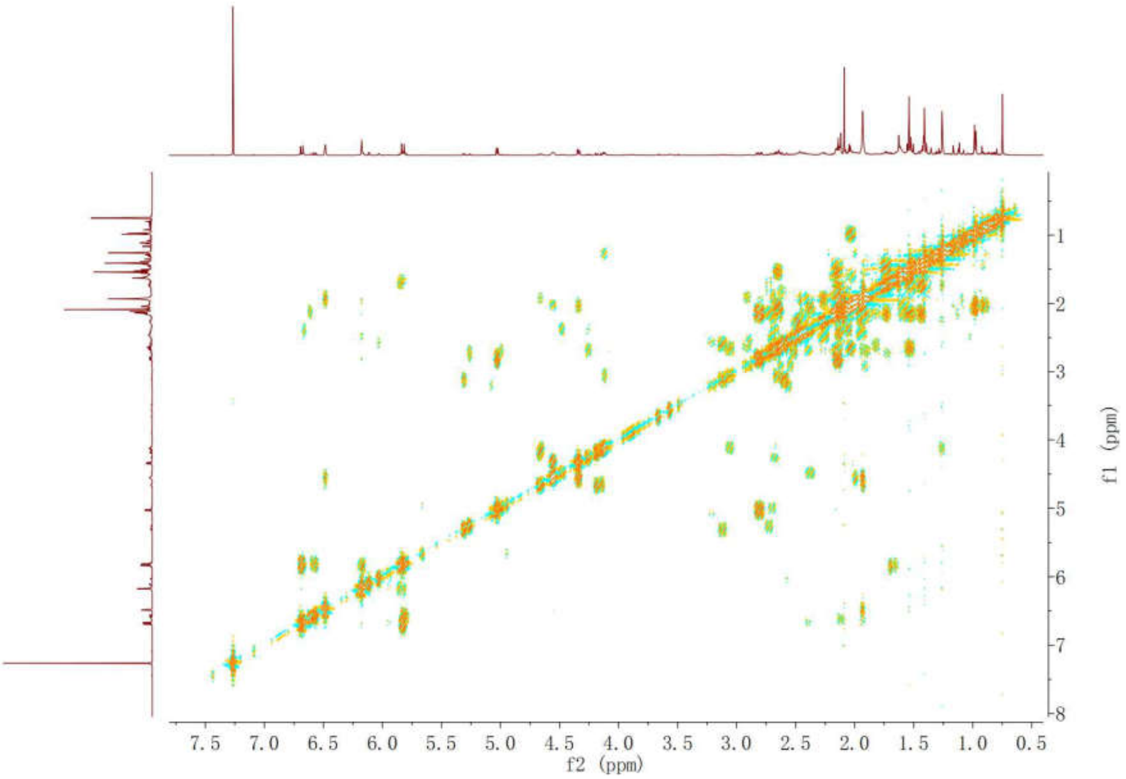

Figure S19. ^{1}H-^{1}H COSY spectrum of compound **3**

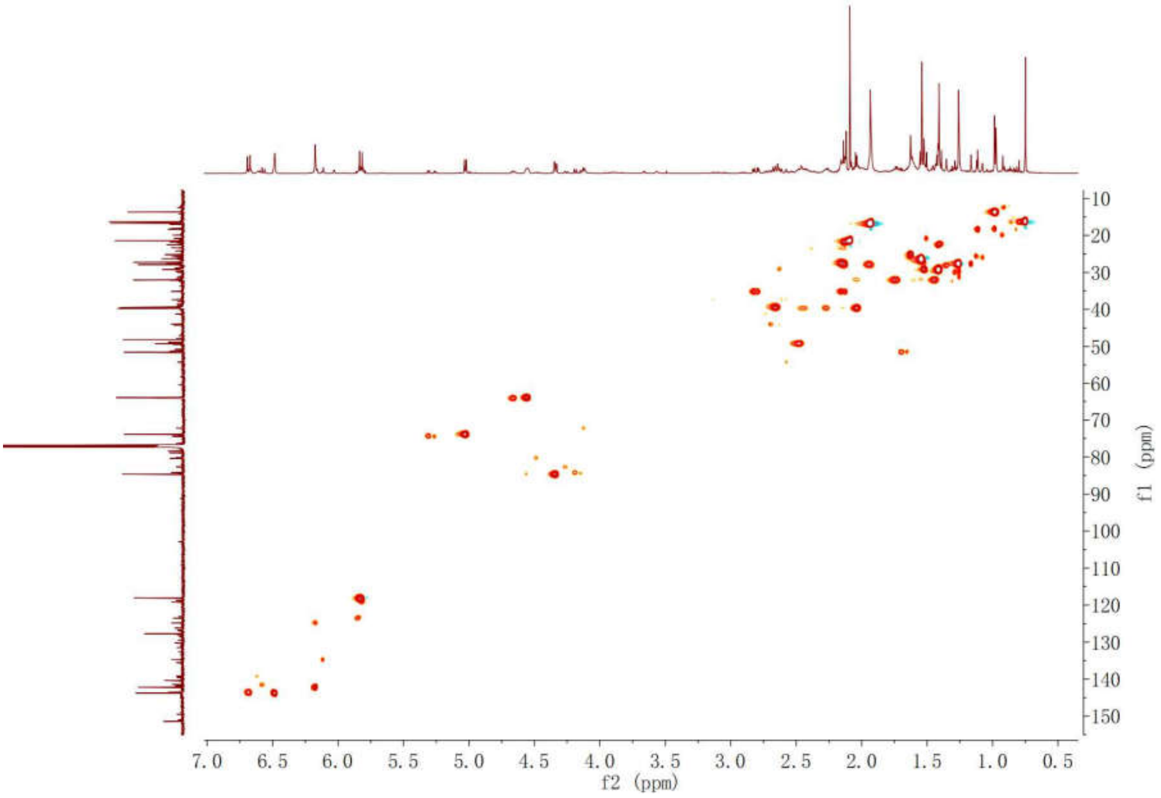

Figure S20. HSQC spectrum of compound **3**

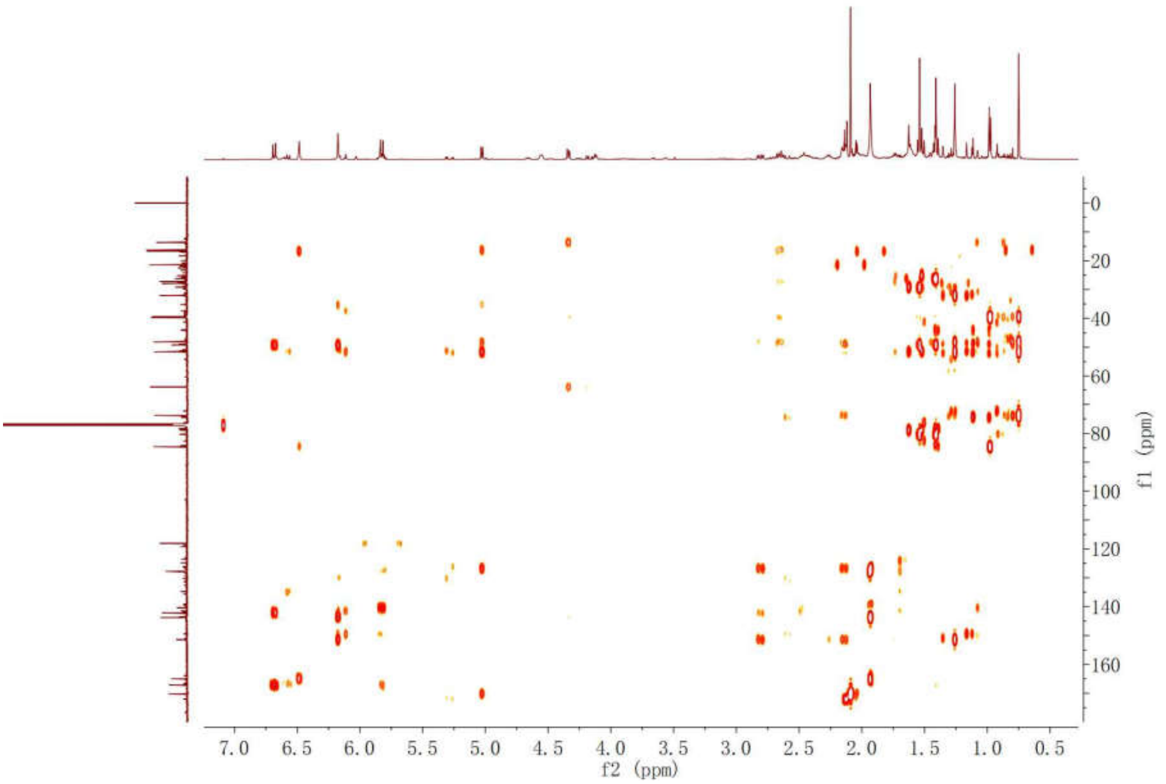

Figure S21. HMBC spectrum of compound **3**

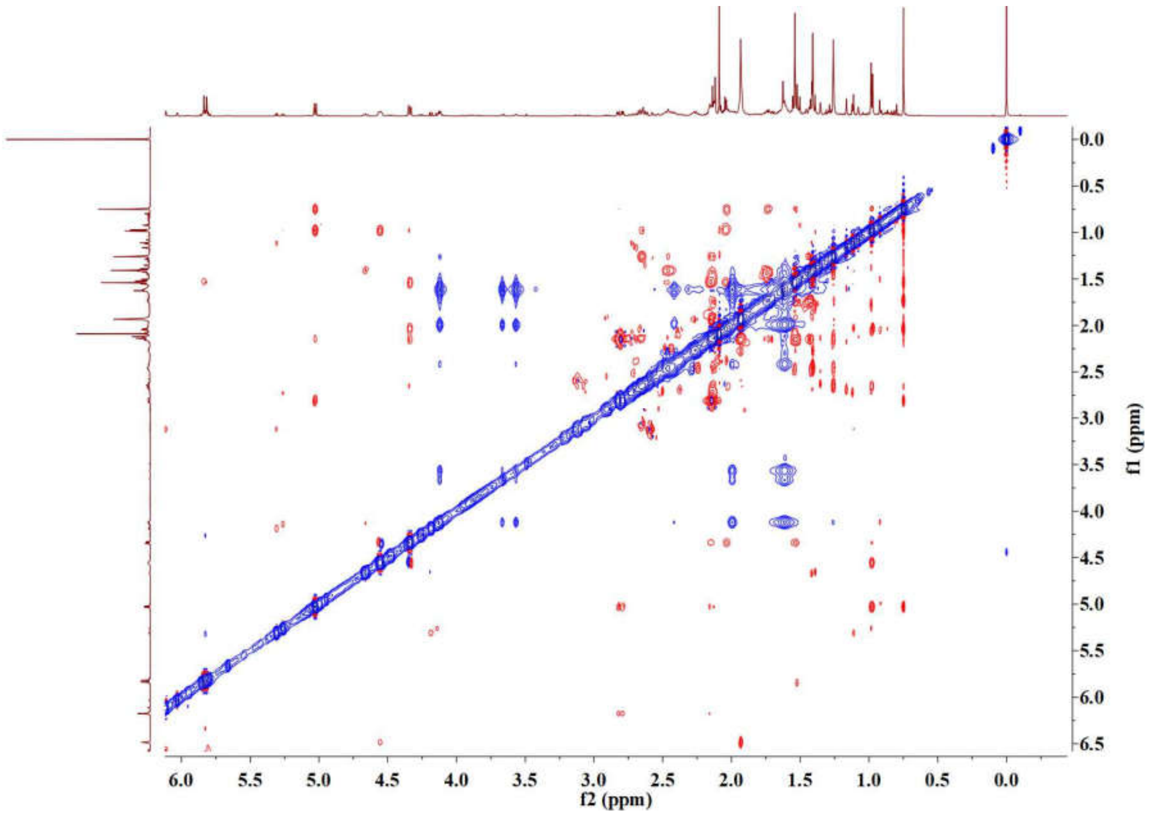

Figure S22. NOESY spectrum of compound **3**

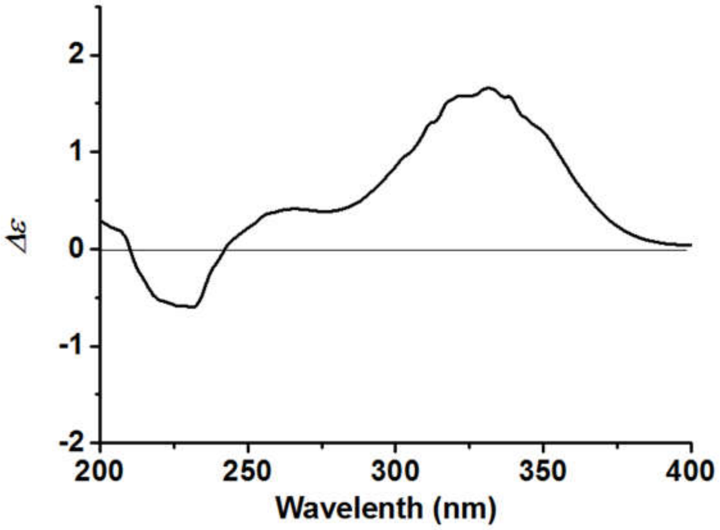

Figure S23. CD spectrum of compound **3**

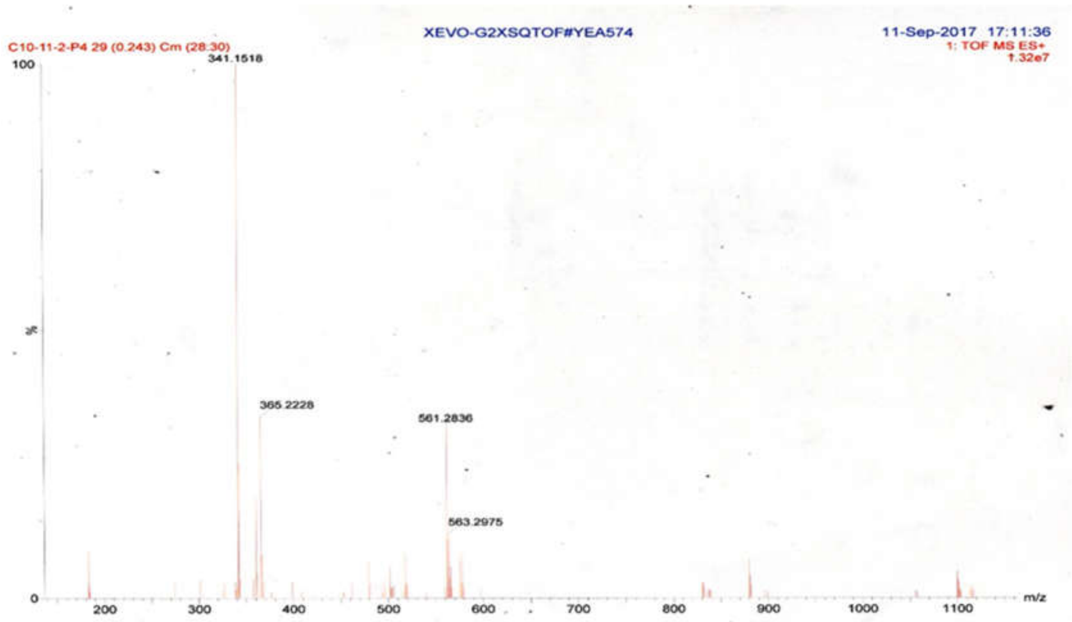

Figure S24. HRESI-MS spectrum of compound **3**

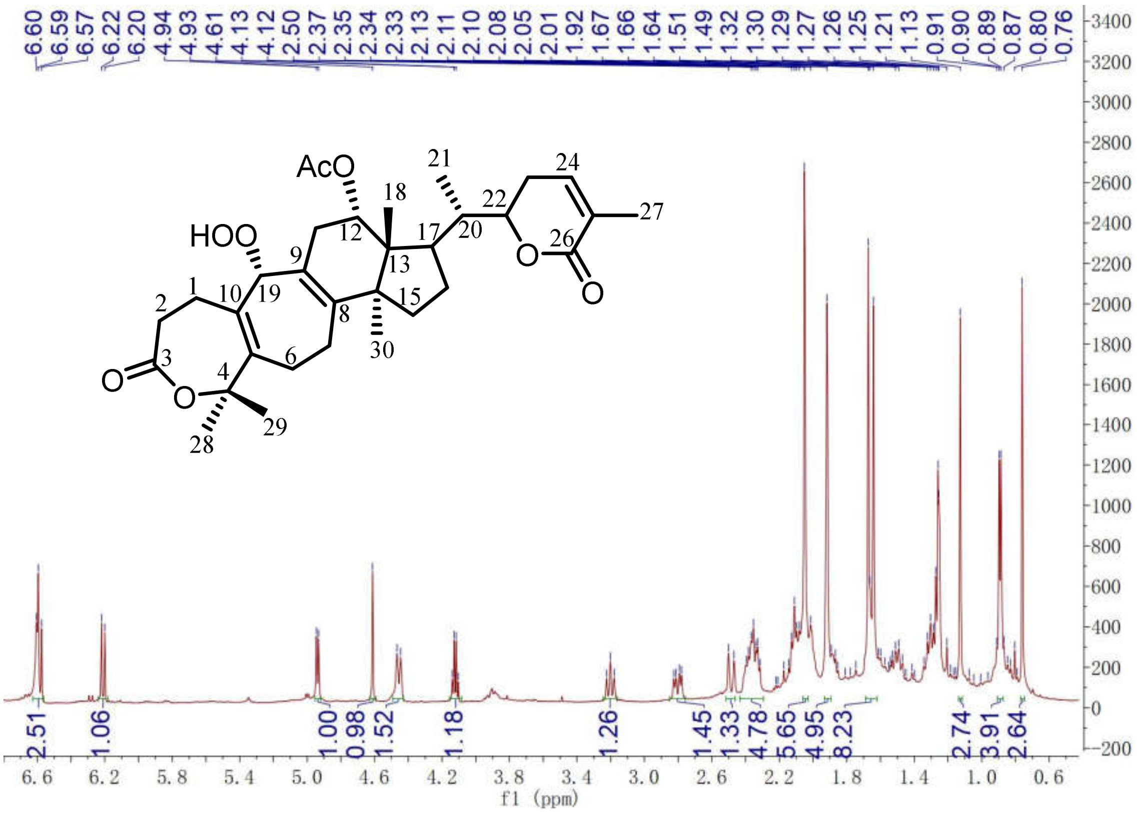

Figure S25. ^{1}H NMR (600 MHz, $CDCl_3$) spectrum of compound **4**

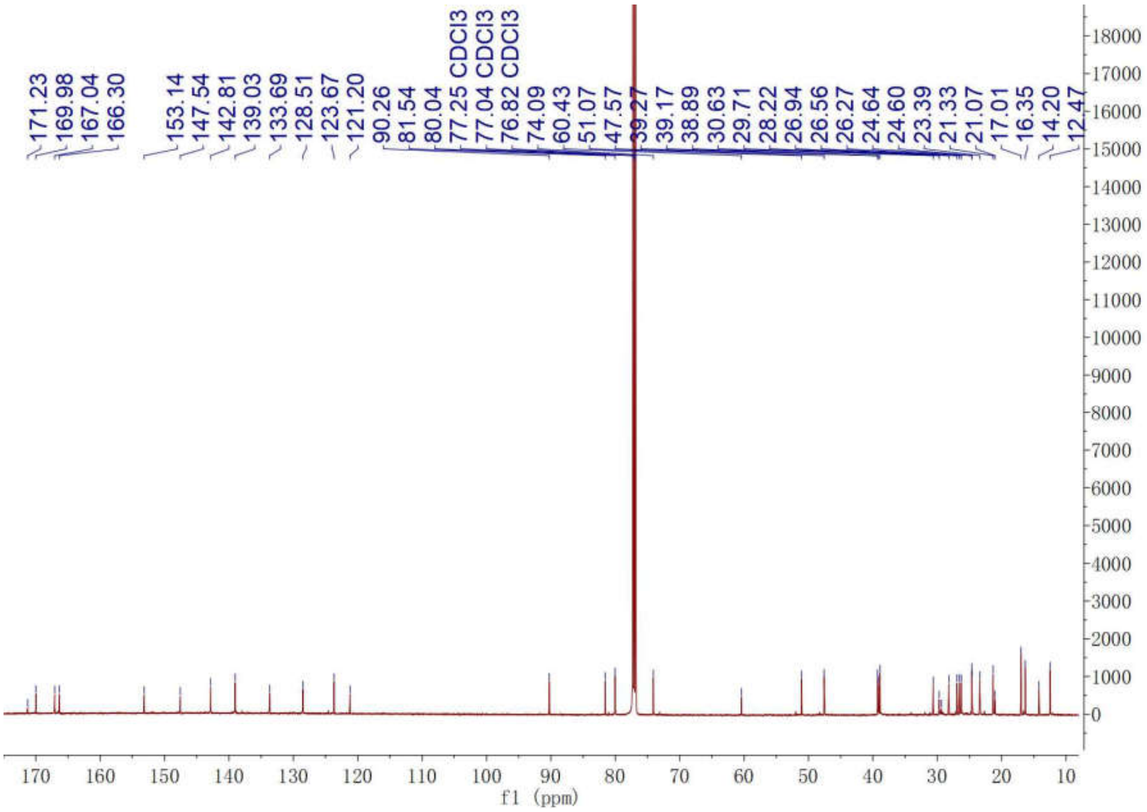

Figure S26. ^{13}C NMR (150 MHz, $CDCl_3$) spectrum of compound **4**

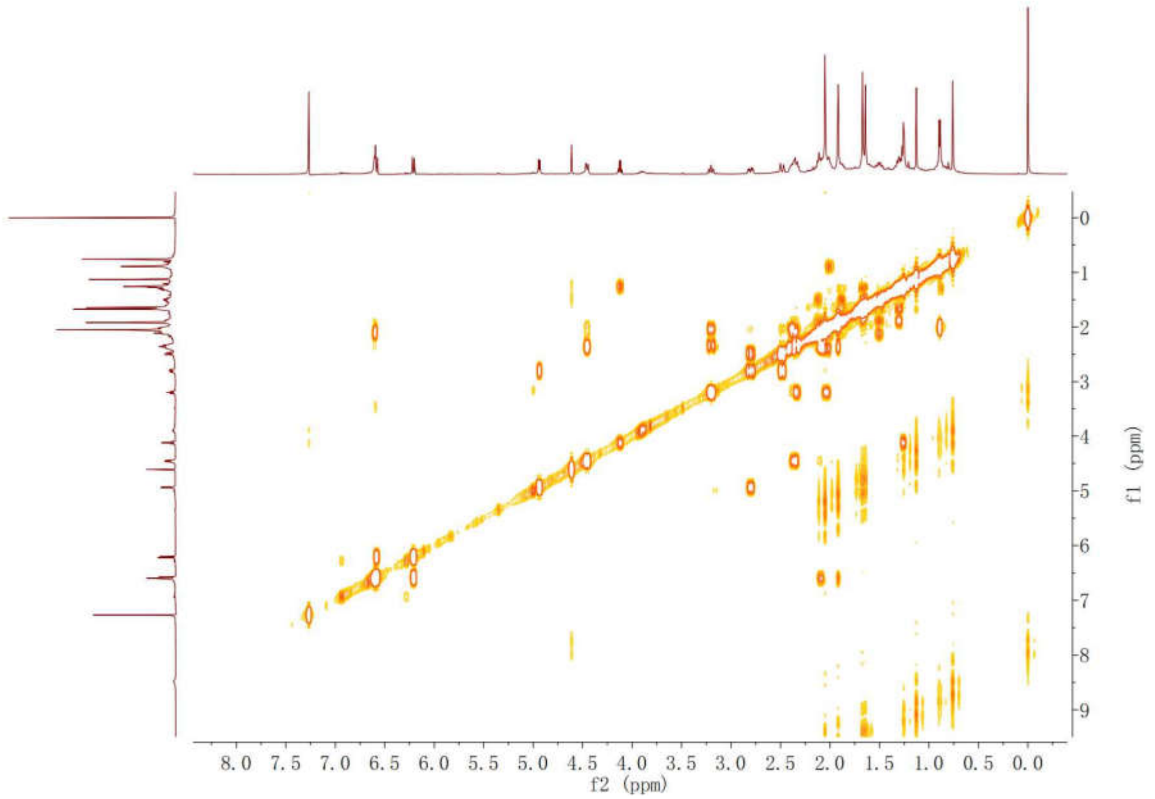

Figure S27. ^{1}H-^{1}H COSY spectrum of compound **4**

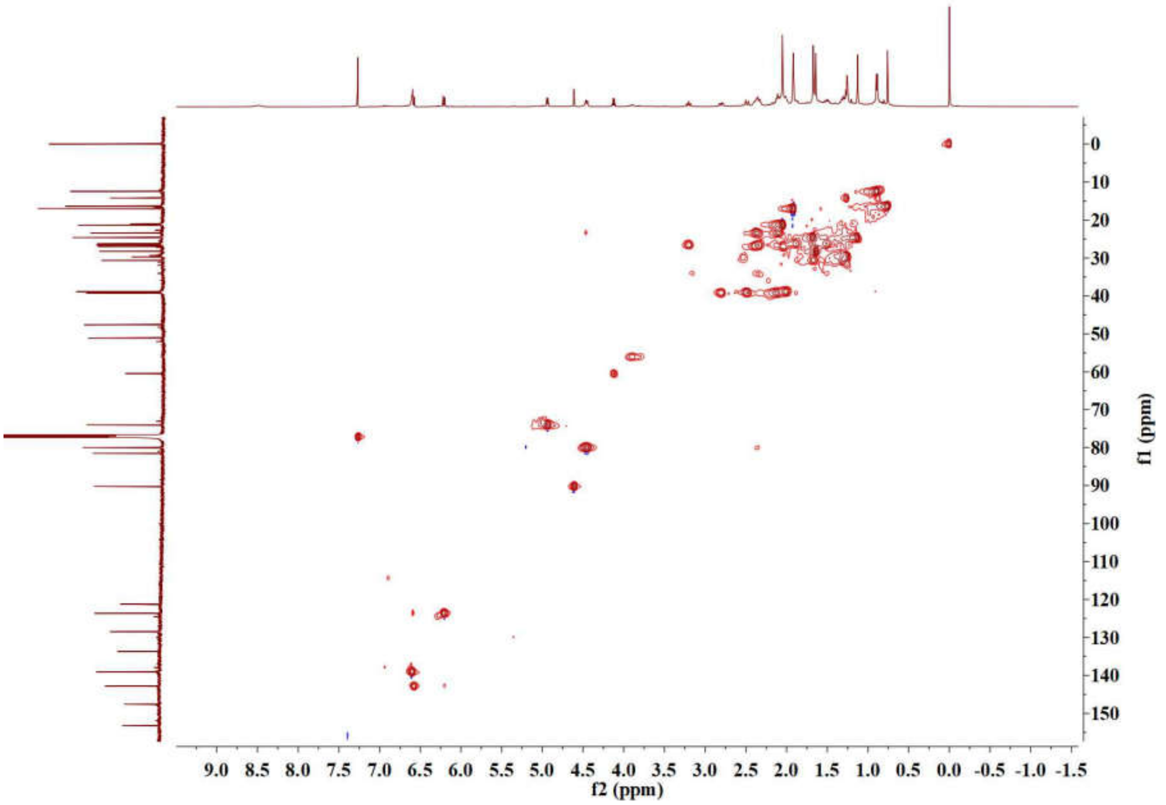

Figure S28. HSQC spectrum of compound **4**

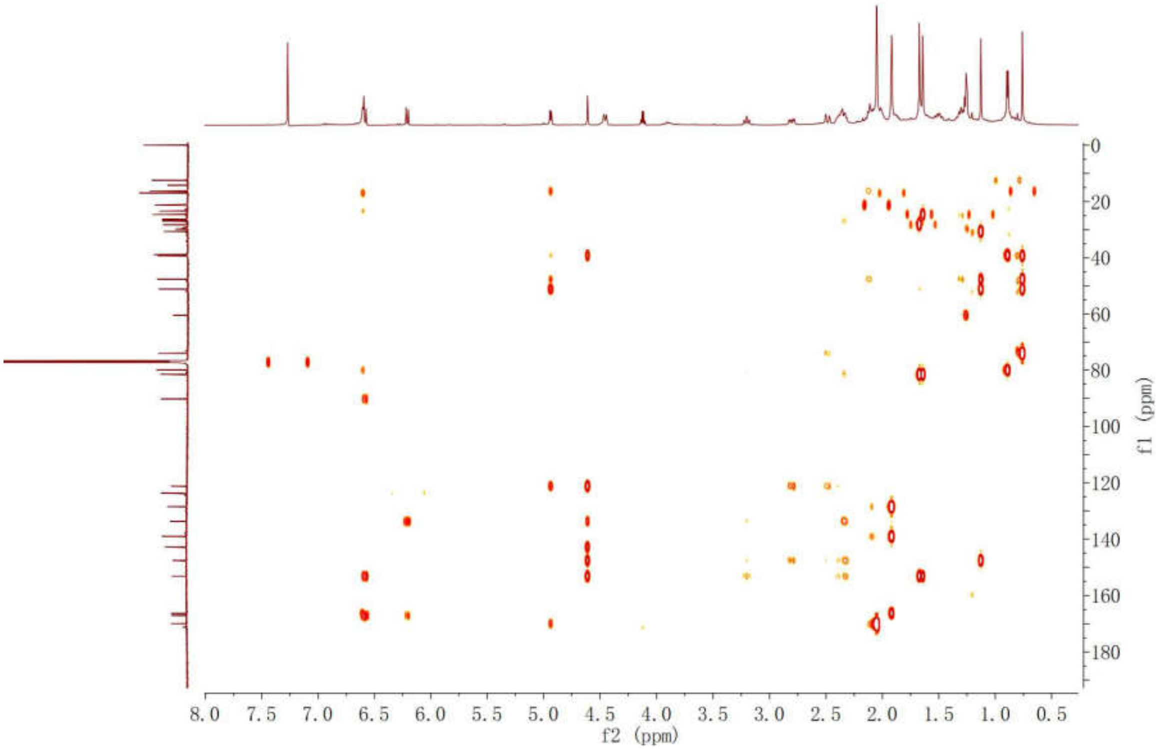

Figure S29. HMBC spectrum of compound **4**

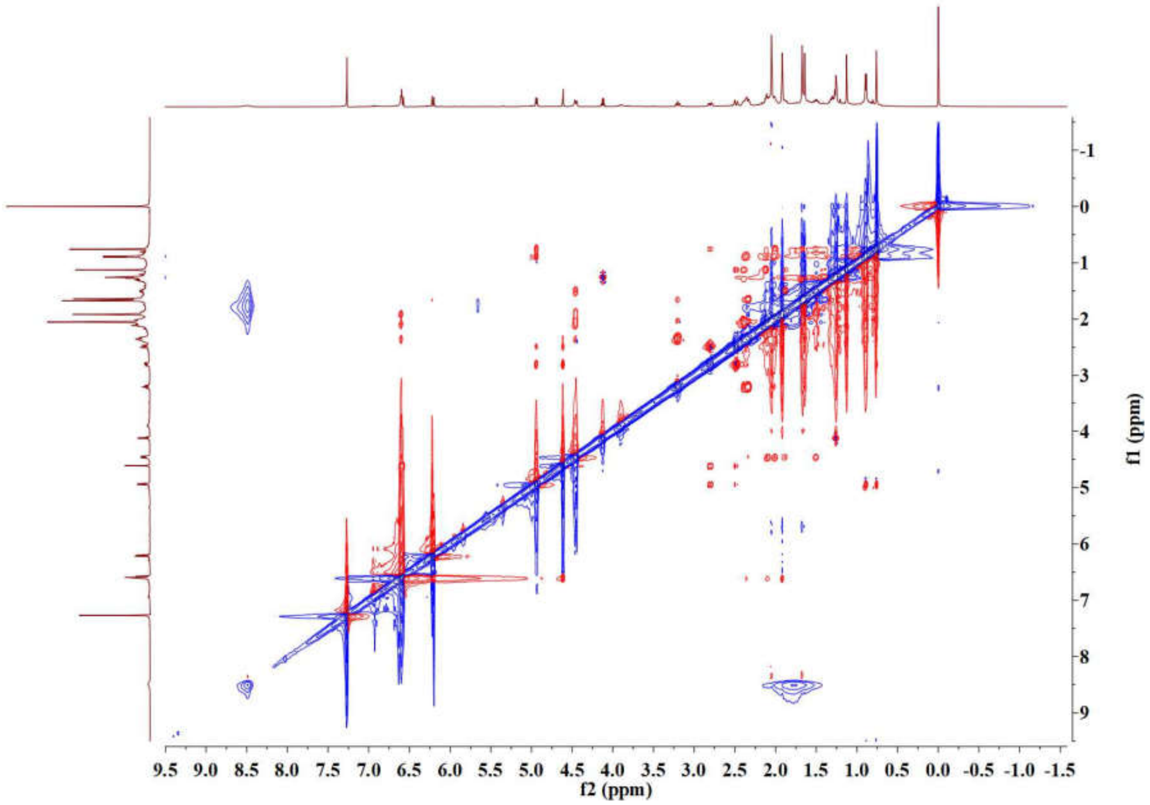

Figure S30. NOESY spectrum of compound **4**

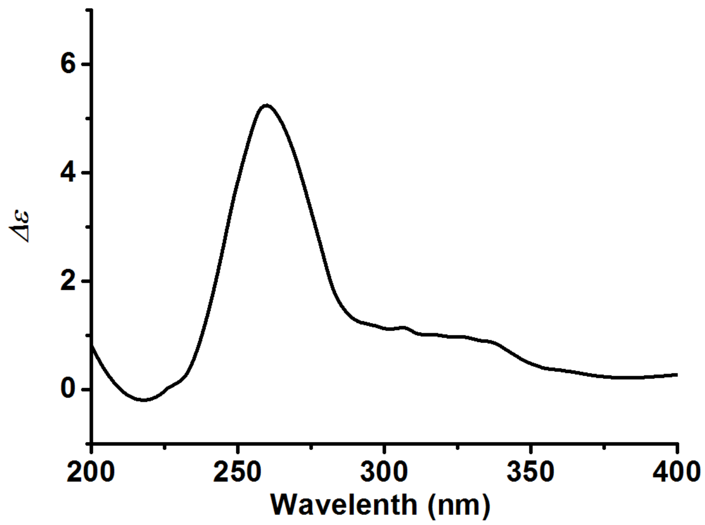

Figure S31. CD spectrum of compound **4**

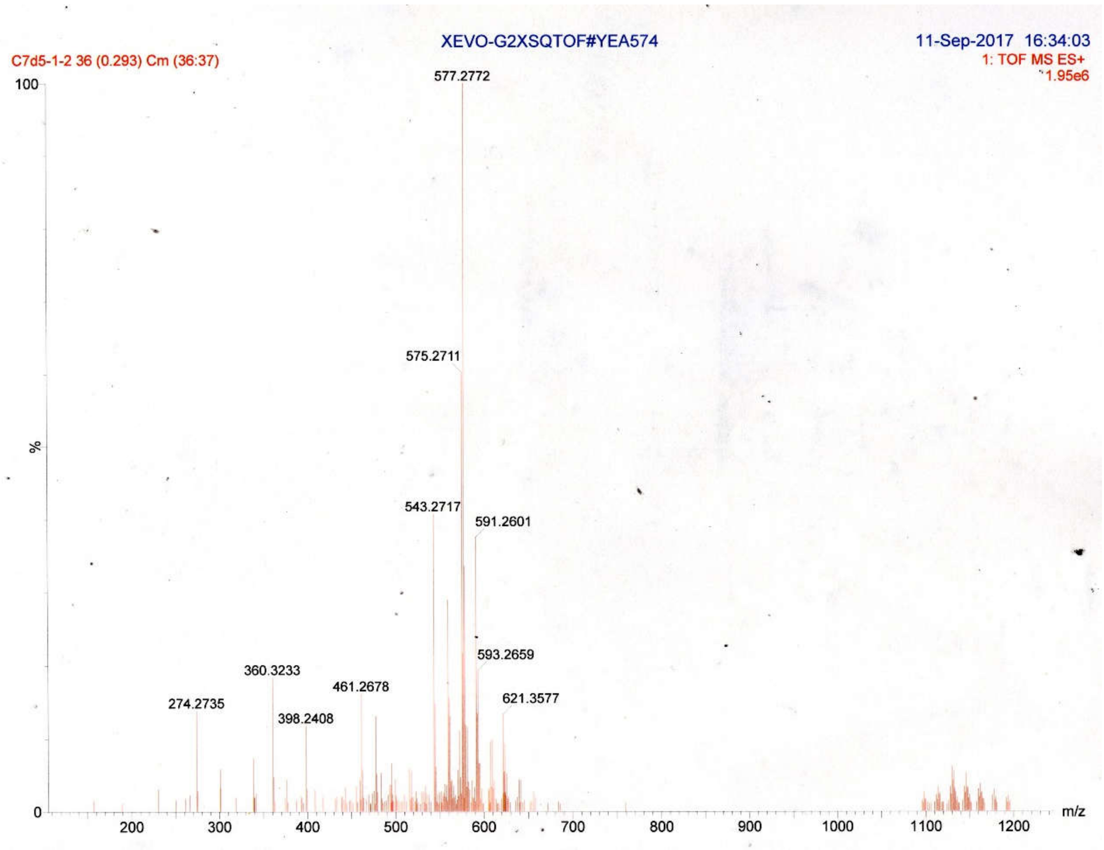

Figure S32. HRESI-MS spectrum of compound **4**

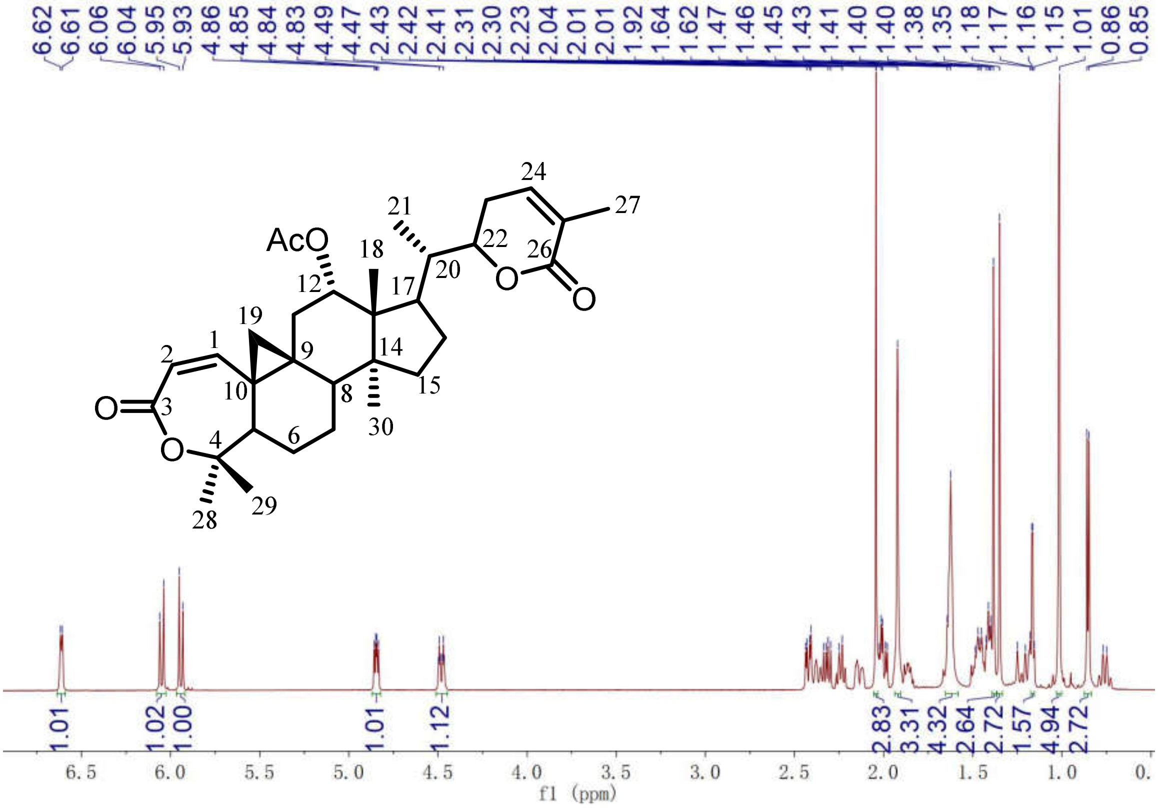

Figure S33. ^{1}H NMR (600 MHz, $CDCl_3$) spectrum of compound **5**

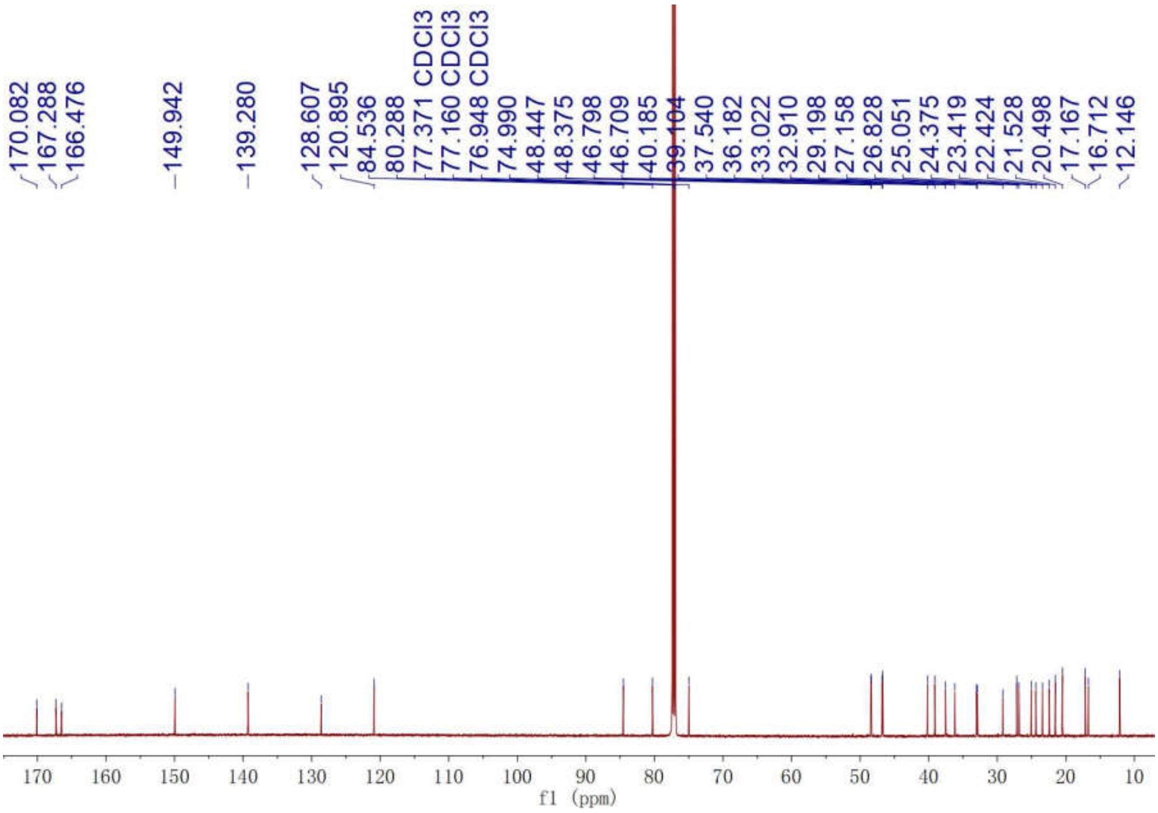

Figure S34. ^{13}C NMR (150 MHz, $CDCl_3$) spectrum of compound **5**

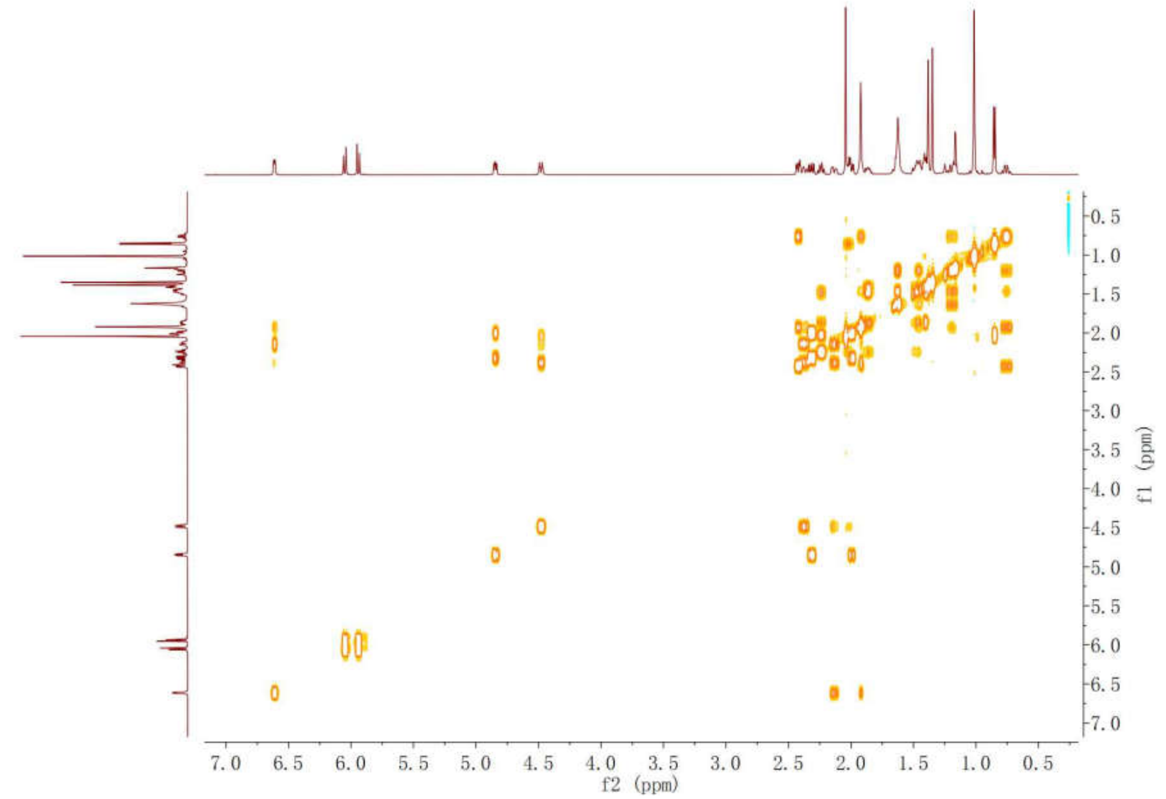

Figure S35. ^{1}H-^{1}H COSY spectrum of compound **5**

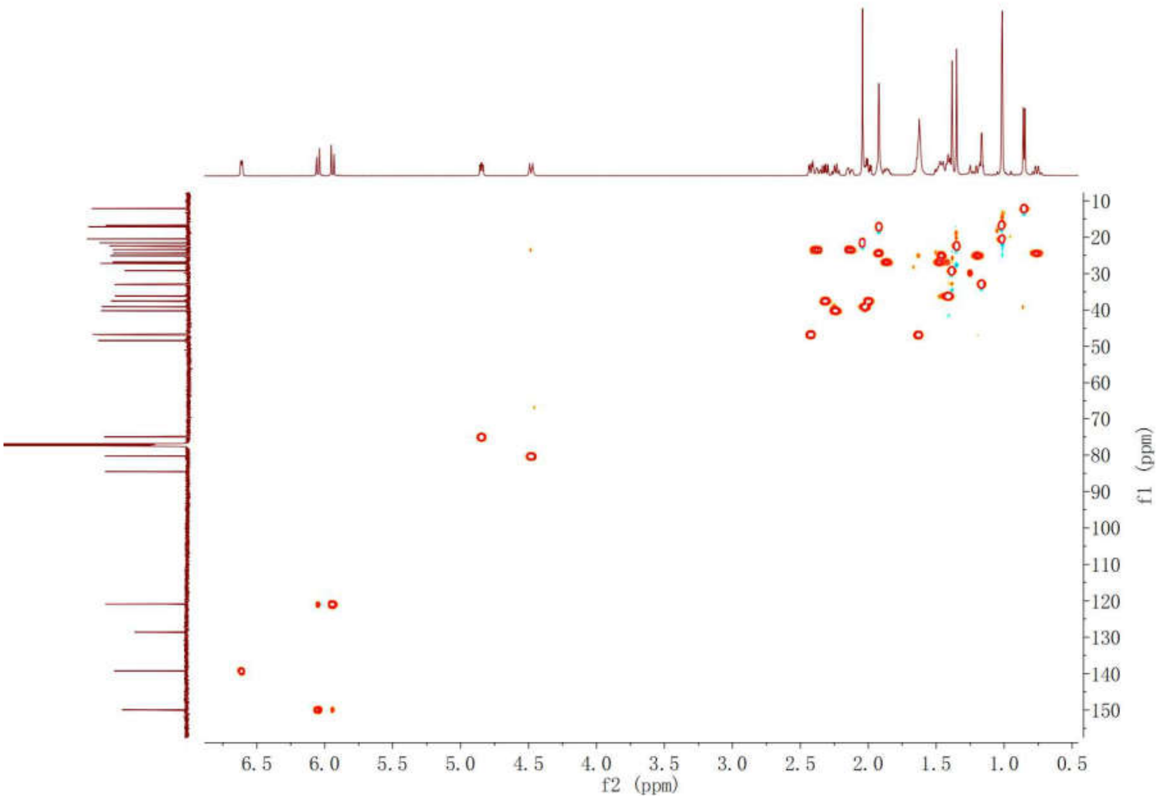

Figure S36. HSQC spectrum of compound **5**

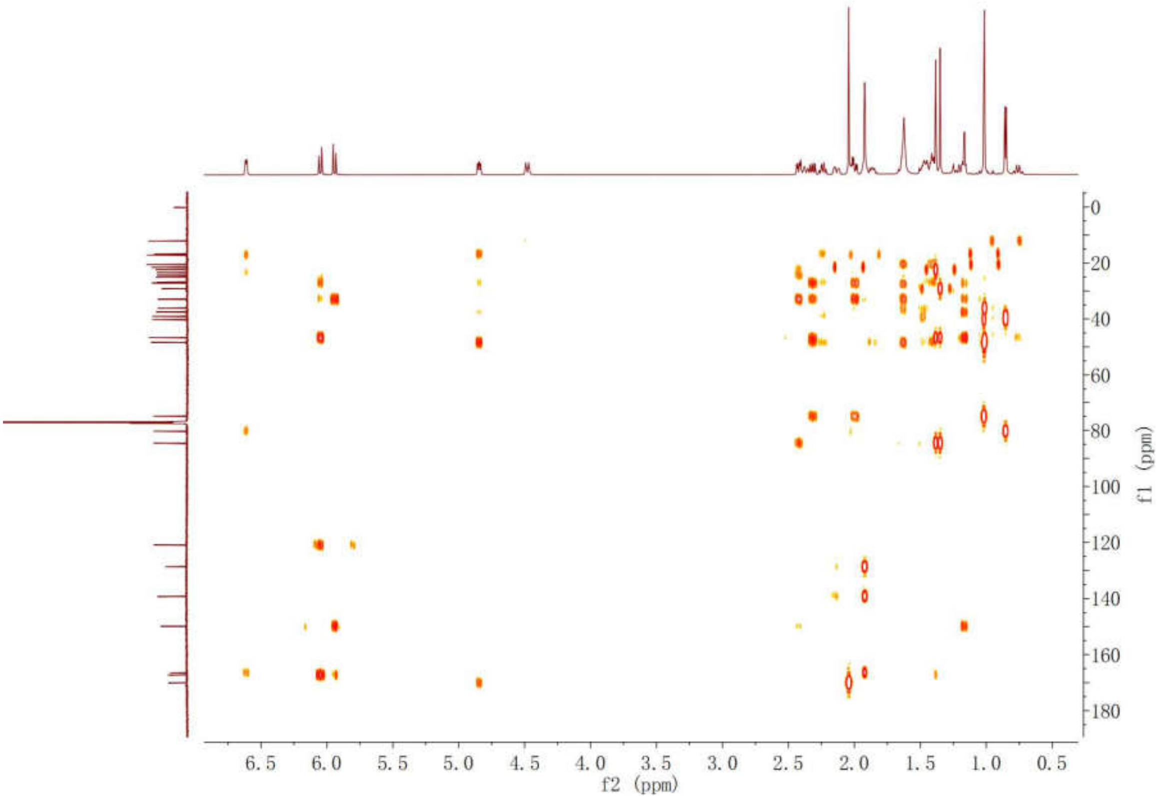

Figure S37. HMBC spectrum of compound **5**

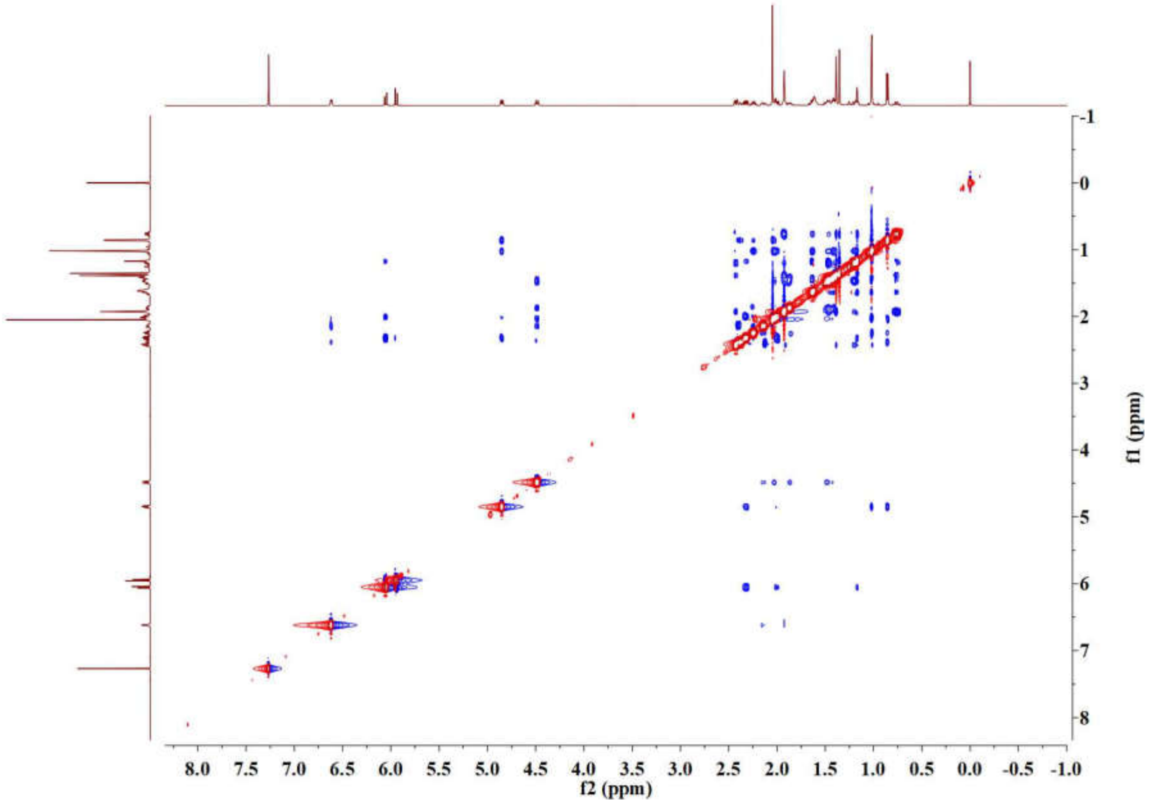

Figure S38. ROESY spectrum of compound **5**

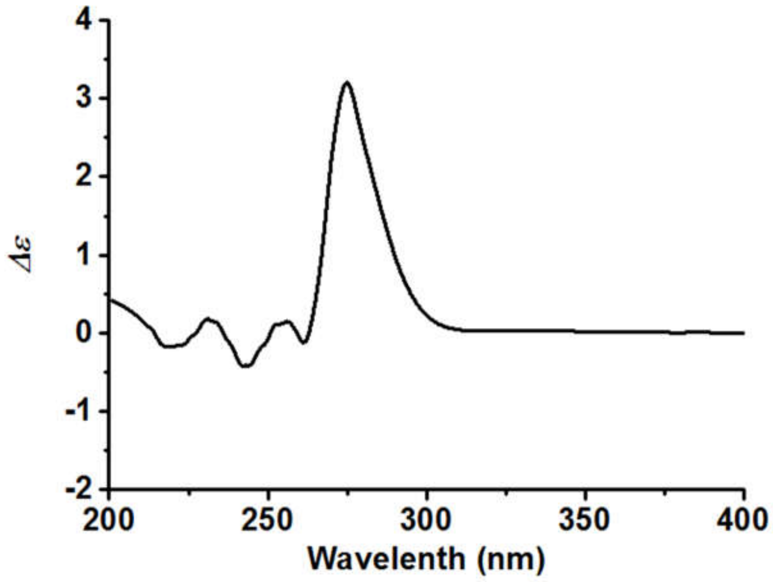

Figure S39. CD spectrum of compound **5**

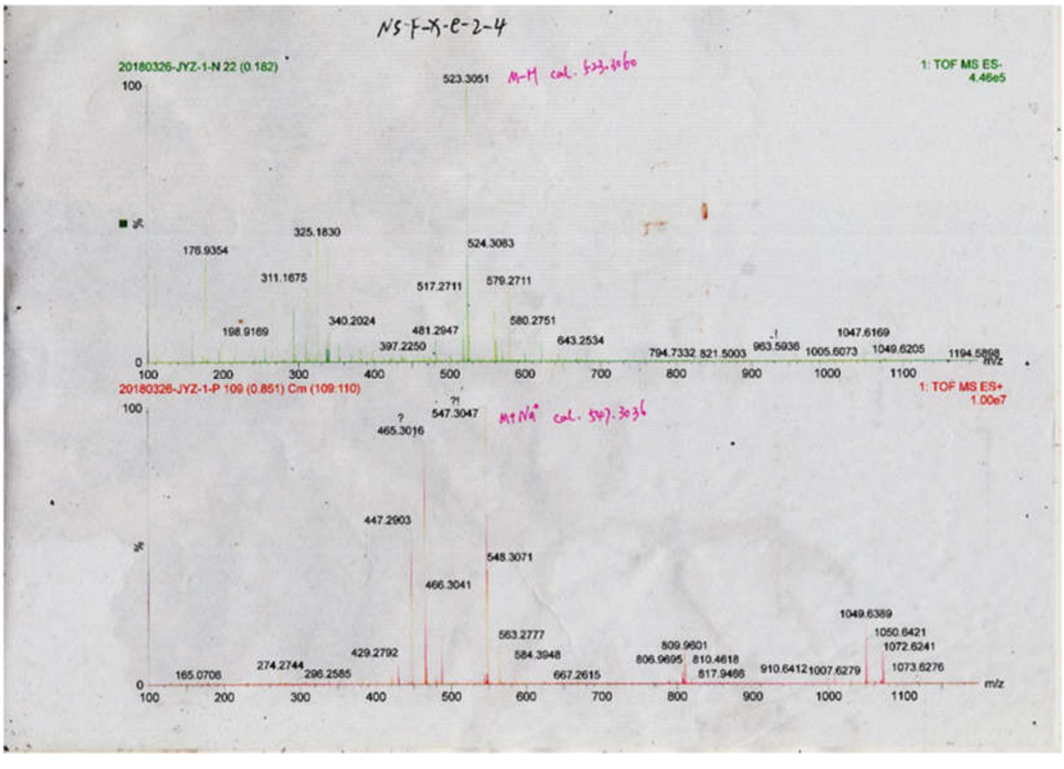

Figure S40. HRESI-MS spectrum of compound **5**

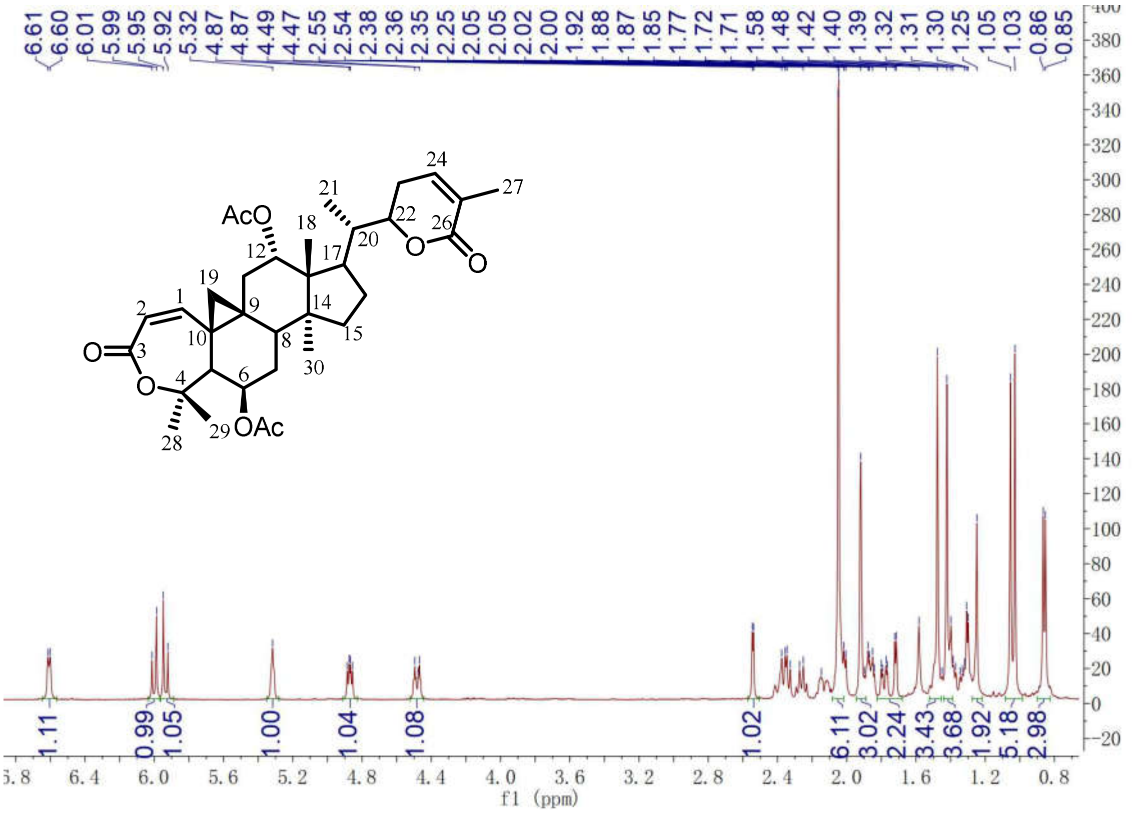

Figure S41. ^{1}H NMR (500 MHz, $CDCl_3$) spectrum of compound **6**

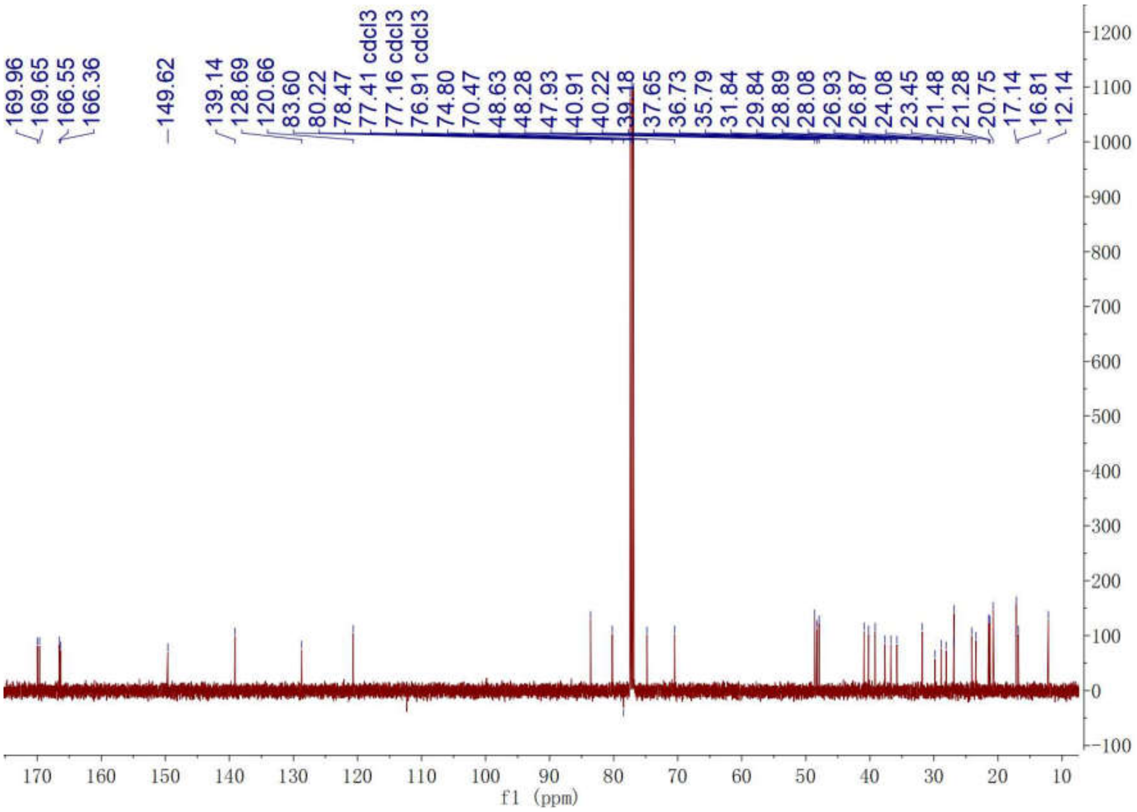

Figure S42. ^{13}C NMR (125 MHz, $CDCl_3$) spectrum of compound **6**

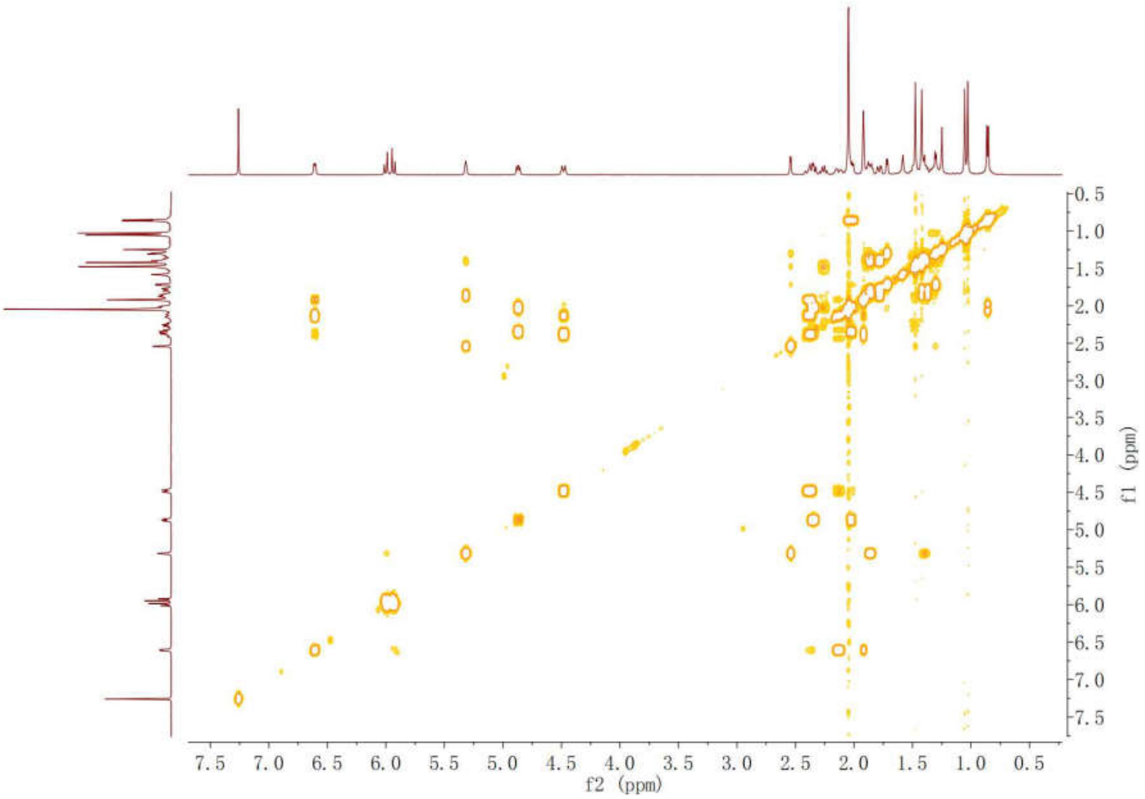

Figure S43. ^{1}H-^{1}H COSY spectrum of compound **6**

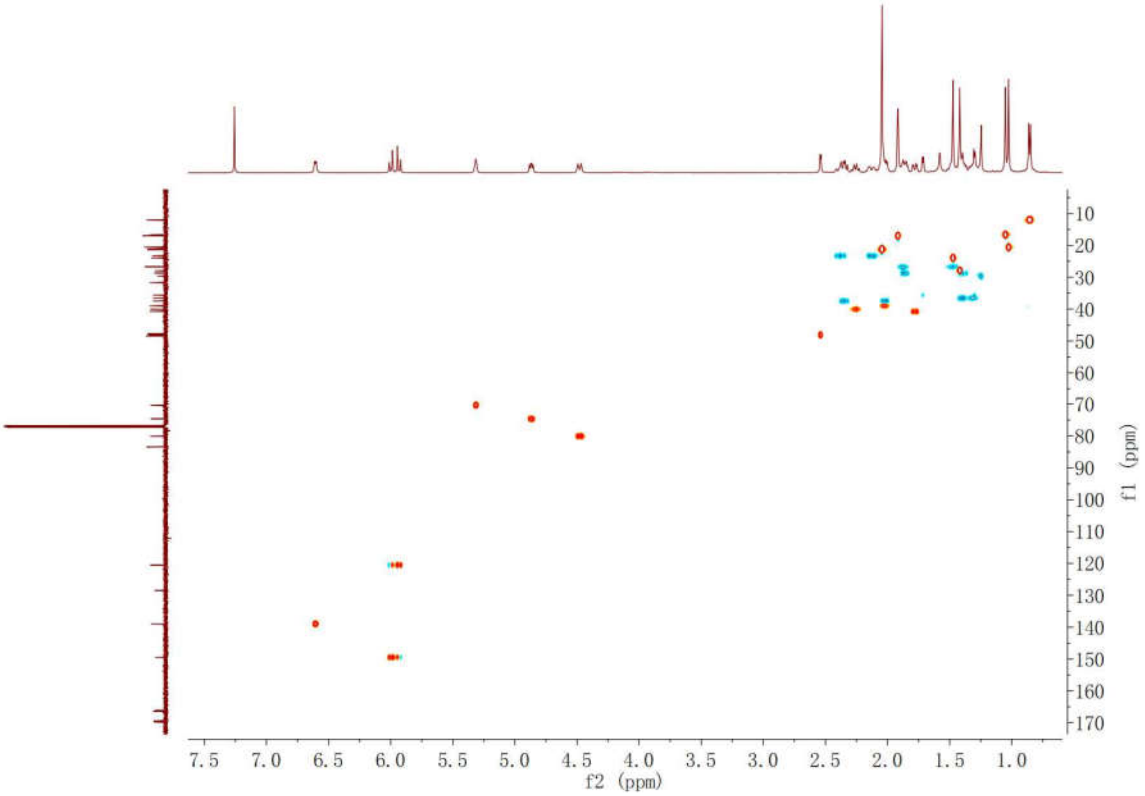

Figure S44. HSQC spectrum of compound **6**

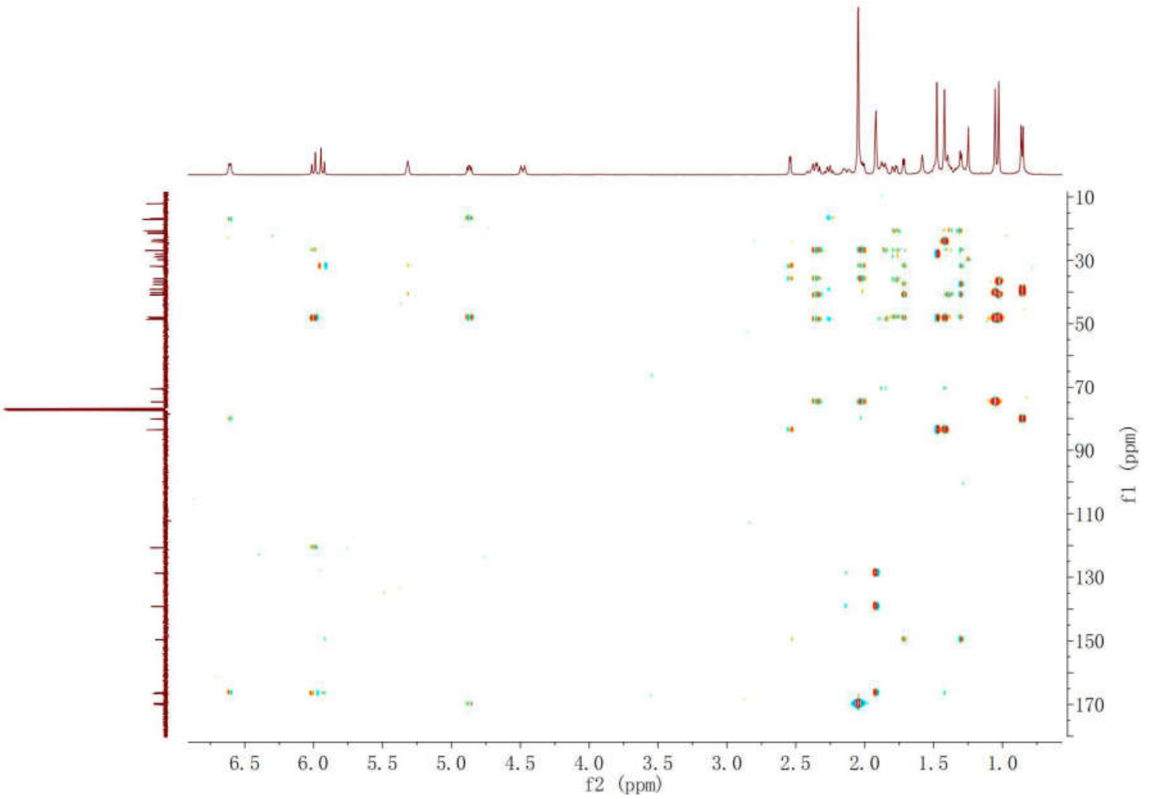

Figure S45. HMBC spectrum of compound **6**

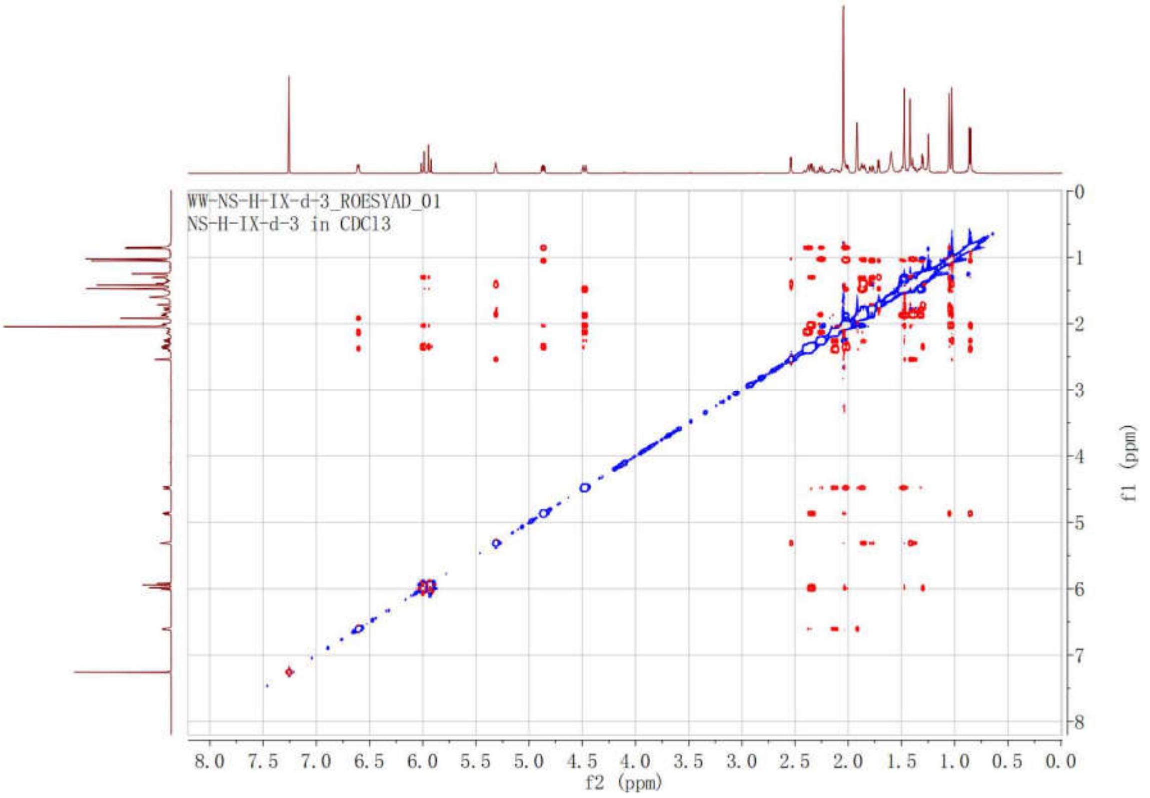

Figure S46. ROESY spectrum of compound **6**

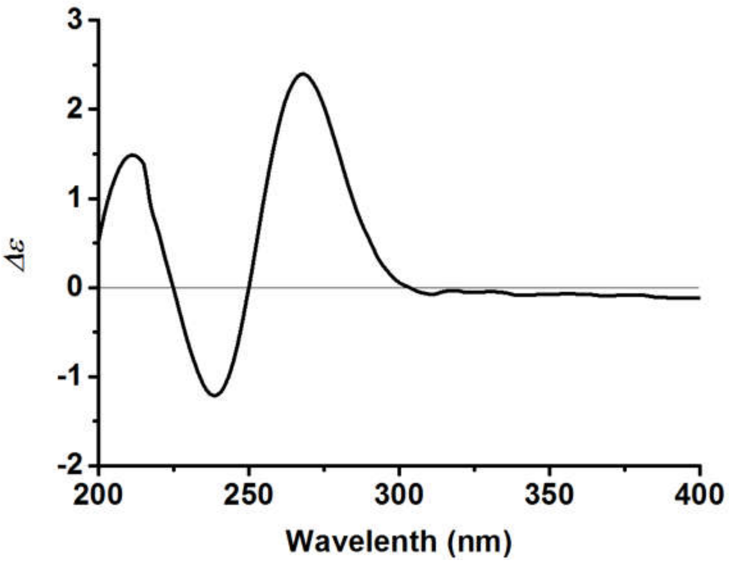

Figure S47. CD spectrum of compound **6**

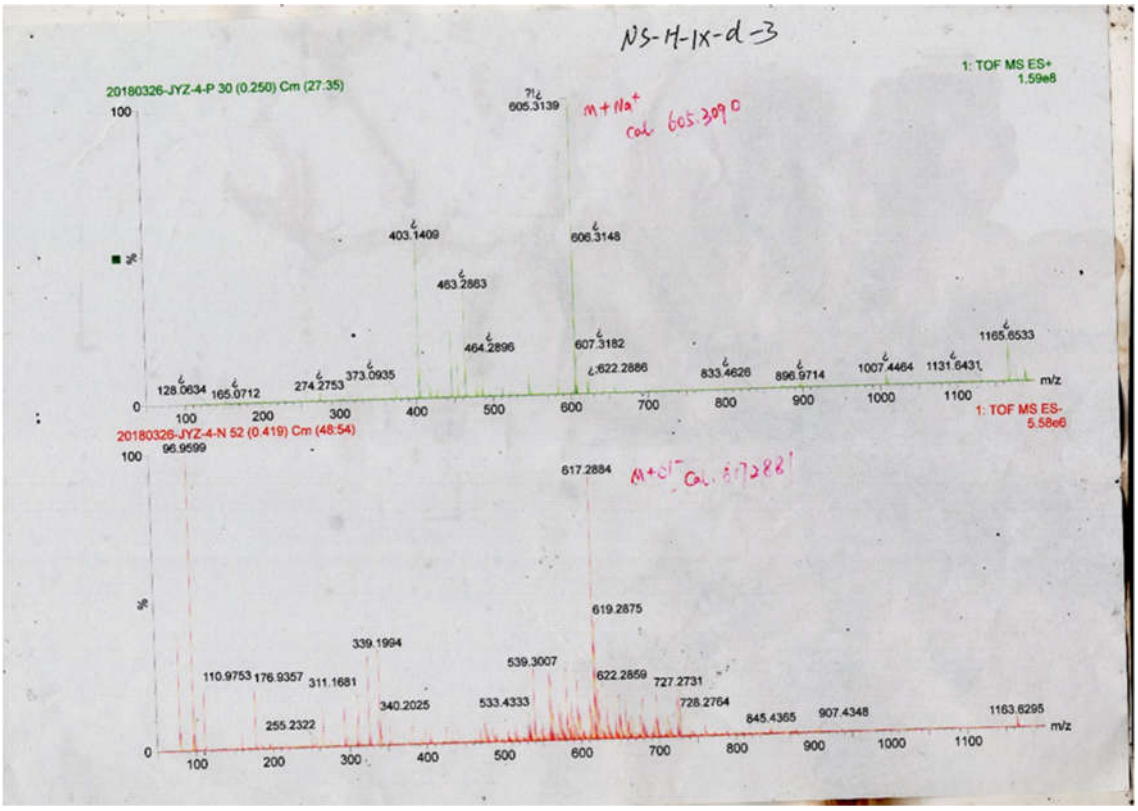

Figure S48. HRESI-MS spectrum of compound **6**
